# Reciprocal costimulatory molecules control the activation of mucosal type 3 innate lymphoid cells during engagement with B cells

**DOI:** 10.1038/s41423-023-01041-w

**Published:** 2023-05-25

**Authors:** Xinping Lv, Shan Zhu, Jing Wu, Jinfeng Shi, Qiuyu Wei, Tete Li, Ning Yang, Chunyan Liu, Lingli Qi, Guoxia Zang, Hang Cheng, Zhiguang Yang, Chengyan Jin, Yusheng Wang, Jiuwei Cui, Hideki Ueno, Yong-Jun Liu, Jingtao Chen

**Affiliations:** 1 Cancer Center, First Hospital of Jilin University, Changchun, Jilin 130021 China; 2 Laboratory for Tumor Immunology, First Hospital of Jilin University, Changchun, Jilin 130061 China; 3 Department of Otolaryngology Head and Neck Surgery, First Hospital of Jilin University, Changchun, Jilin 130021 China; 4 Department of Translational Medicine, Changchun GeneScience Pharmaceuticals Co., Ltd., Changchun, Jilin 130012 China; 5 Department of Gynecology, First Hospital of Jilin University, Changchun, Jilin 130021 China; 6 Department of Pediatric Gastroenterology, First Hospital of Jilin University, Changchun, Jilin 130021 China; 7 Department of Pediatrics, First Hospital of Jilin University, Changchun, Jilin 130021 China; 8 Department of Thoracic Surgery, First Hospital of Jilin University, Changchun, Jilin 130021 China; 9 Department of Thoracic Surgery, Second Hospital of Jilin University, Changchun, Jilin 130041 China; 10 Department of Immunology, Graduate School of Medicine, Kyoto University, Kyoto, Japan; 11 ASHBi Institute for the Advanced Study of Human Biology, Kyoto University, Kyoto, Japan

**Keywords:** Innate lymphoid cell, B cell, Tonsil, ICOS, Costimulatory molecule, Innate lymphoid cells, Interleukins, Mucosal immunology, Tonsils

## Abstract

Innate lymphoid cells (ILCs) are the counterpart of T helper cells in the innate immune system and share multiple phenotypes with T helper cells. Inducible T-cell costimulator (ICOS) is recognized on T cells and participates in T-cell activation and T and B-cell engagement in lymphoid tissues. However, the role of ICOS in ILC3s and ILC3-involved interactions with the immune microenvironment remains unclear. Here, we found that ICOS expression on human ILC3s was correlated with the activated state of ILC3s. ICOS costimulation enhanced the survival, proliferation, and capacity of ILC3s to produce cytokines (IL-22, IL-17A, IFN-γ, TNF, and GM-CSF). Via synergistic effects of ICOS and CD40 signaling, B cells promoted ILC3 functions, and ILC3-induced T-cell-independent B-cell IgA and IgM secretion primarily required CD40 signaling. Hence, ICOS is essential for the nonredundant role of ILC3s and their interaction with adjacent B cells.

## Introduction

Innate lymphoid cells (ILCs) are heterogeneous lineage^neg^ innate immune cells that have emerged in the past 10 years. As the innate counterparts of T helper cells, ILCs are divided into ILC1s, ILC2s, and ILC3s; the different ILCs have different transcription factor requirements and cytokine-producing capacities, resulting in their involvement in different types of immune responses, especially in mucosal immunity. Among the three major subsets, ILC3s produce a large amount of lineage-specific interleukin (IL)-22, IL-17, and other products, such as granulocyte macrophage colony-stimulating factor (GM-CSF). As a result of their polyreactive phenotype such as major histocompatibility complex ΙΙ (MHCΙΙ) [1, 2], Toll-like receptors [3], and nonspecific molecules, such as B-cell activation factor (BAFF) [4, 5], ILC3s impact multiple immune cells in the microenvironment directly and indirectly. However, studies on the interaction between ILC3s and other immune cells, in particular the impact of the immune microenvironment on ILC3s, are still insufficient, and their results are unclear.

Inducible T-cell costimulator (ICOS) is primarily expressed on activated T cells and belongs to the CD28 coreceptor family [6]. ICOS ligand (ICOSL) is expressed on various immune and nonimmune cells, including B cells, dendritic cells (DCs), macrophages, and epithelial cells [7]. The pattern of ICOS and ICOSL expression determines the diversity of intercellular interactions initiated by ICOS and ICOSL interaction. For example, the ICOS and ICOSL interaction can not only mediate plasmacytoid DC (pDC)-induced IL-10 production by Foxp3^+^ T cells [8] but also promote follicular T helper (Tfh) cells and the B-cell response in lymphoid tissues [9, 10]. Since ILCs were discovered, studies have identified the expression of ICOS [11, 12] and ICOSL [11] on ILC2s, wherein it affects ILC2 homeostasis and the production of IL-5 and IL-13 [11]. Recently, ICOS has been detected on the surface of human ILC3s [13]; however, its role in ILC3 biology has not been extensively evaluated. Therefore, investigating the influence of ICOS on ILC3s and the specific role of ICOS signaling in mediating the crosstalk between ILC3s and the immune microenvironment is of great importance for the study of ILC3 immunological characteristics and the role of ILC3s in mucosal immunity.

ILC3s regulate B-cell homeostasis and function in different tissues [5]. In a T-cell–independent manner, human retinoic acid receptor-related orphan receptor-γt (RORγt)^+^ ILCs activate naïve B, marginal zone B, and plasma cells by expressing BAFF, CD40L, and Delta-like 2, a Notch ligand [4, 5], further inducing IgA-switching in lymphoid structures [14, 15]. Through a T-cell-dependent process, ILC3s present antigens to Tfh cells to regulate IgA switching in B cells within gut-associated lymphoid tissues [16]. In general, the high colocalization with B cells, complicated phenotype, and tissue-resident characteristics of ILC3s jointly underly the close relationship between ILC3s and B cells. However, because of the low proportion and absolute count of ILC3s, especially in humans, the influence of B cells on ILC3s and the mechanism of their interaction are still not thoroughly understood. Because B cells were the first recognized ICOSL-expressing cells [17] and considering the expression of ICOS on ILC3s, it is of great importance to explore the role of the ICOS/ICOSL pathway in the interaction between ILC3s and B cells.

Here, we found that ICOS was primarily expressed on ILC3s in human solid tissues, and ICOS expression was upregulated after stimulation with IL-7 + IL-2 + IL-1β + IL-23. ICOS stimulation by ICOSL improved the survival, proliferation, and activation of ILC3s, including increasing IL-22 and GM-CSF production and enhancing CD69 expression. ILC3 and B-cell interaction promoted ILC3 and B-cell survival and proliferation and facilitated the production of IL-22 by ILC3s and the secretion of immunoglobulins by B cells. Moreover, the effect of B cells on ILC3s was synergistically mediated by the ICOS and CD40 pathways, and the ILC3 support of B cells primarily required CD40 signaling. Thus, this study reveals the unique role of ILC3s in the mucosal immune system and the regulatory mechanism connecting ILC3s and adaptive immune cells.

## Results

### Identification of costimulatory molecule ICOS expression on human ILC3s

**Fig. 1:**
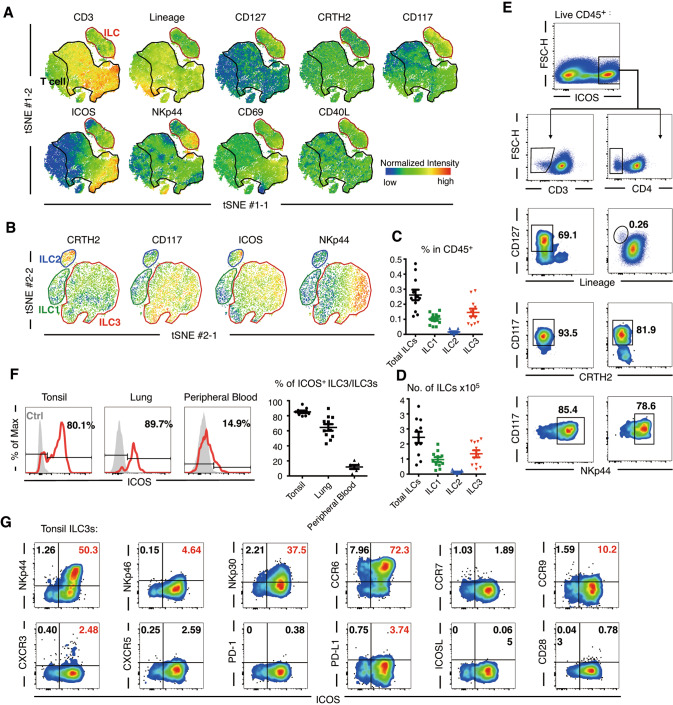
ICOS expression is not limited to T cells and ILC2s. **A**, **B** Corresponding to the gating strategy in Supplementary Fig. 1a in detail; t-distributed stochastic neighbor embedding (t-SNE) analysis was performed using live tonsillar CD45^+^ cells. **A** Using identified biomarker sets, the T-cell cluster was manually gated as CD3^+^ cells (black), and the cluster of total innate lymphoid cells (ILCs) was identified as lineage (CD3 CD19 CD20 CD14 CD94 CD34 CD1a CD11c CD123 TCRα/β TCRγ/δ FcεRIα)^−^ CD127^+^ (red). **B** In ILCs, a second t-SNE analysis was conducted to focus on three clusters corresponding to ILC1s (green), ILC2s (blue), and ILC3s (red). Normalized intensities of each biomarker were calculated and displayed on the plot. The results correspond to three donors. Frequencies (**C**) and cell counts (per gram) (**D**) of total ILCs (lineage^−^ CD127^+^), ILC1s (lineage^−^ CD127^+^ CRTH2^−^ CD117^−^), ILC2s (lineage^−^ CD127^+^ CRTH2^+^), and ILC3s lineage^−^ CD127^+^ CRTH2^−^ CD117^+^) from gated sets of live tonsillar CD45^+^ cells are shown in FACS dot plots (n = 11). **E** The inducible T-cell costimulator (ICOS)^high^ lymphocytes were gated and indicated as CD3^−^ or CD4^−^ ILC3s. **F** ICOS expression of ILC3s (left) and frequencies of ICOS^+^ ILC3s in ILC3s from human tonsil, distal lung sample (more than 5 cm from the tumor lesion) from donors with lung cancer, and peripheral blood of healthy donors (*n* = 5–11). **G** Coexpression of ICOS and common biomarkers on tonsillar ILC3s. The error bars indicate the mean ± standard error of the mean (SEM)

To investigate ICOS expression on ILC subgroups, flow cytometry analysis combined with t-distributed stochastic neighbor embedding (t-SNE) analysis of lymphocytes isolated from noninflamed palatine tonsils was applied; the results showed that ILC3s were the predominant population within tonsillar ILCs (Fig. 1A–D). Despite containing distinctive clusters, tonsillar ILCs (lineage^−^ CD127^+^) shared some characteristics with tonsillar T cells, including partial expression of ICOS and CD69 as well as low expression of CD40L (Fig. 1A). In contrast, the natural cytotoxicity receptor (NCR) NKp44 was expressed on ILCs but not on T cells (Fig. 1A). Among the three defined ILC subgroups, ICOS expression was detectable on ILC3s (lineage^−^ CRTH2^−^ CD117^+^), some ILC1s (lineage^−^ CRTH2^−^ CD117^−^), and some cells resembling ILC2s (lineage^−^ CRTH2^+^ CD117^−^) as previously reported [11] (Fig. 1B, Supplementary Fig. 1a–c). Additionally, ICOS^high^ tonsillar lymphocytes contained not only CD3^+^ and CD4^+^ cells but also a certain population of ILC3-like cells (Fig. 1E); furthermore, the percentage of ICOS^+^ ILC3s in the tonsil and lung was generally higher than that in peripheral blood (PB) (Fig. 1F), and this higher expression was accompanied by the expression of the ILC3 activation-related biomarker NKp44 [18, 19] (Supplementary Fig. 1d).

Some known activation-associated molecules were detected on ICOS^+^ ILC3s but to a lesser extent on ICOS^−^ ILC3s (Fig. 1G). However, the expression of CD28 and the conventional checkpoint PD-1 was low on tonsillar ILC3s, even on ICOS^+^ ILC3s (Fig. 1G). In contrast to ILC2s [11], ILC3s lacked ICOSL expression (Fig. 1G). Collectively, the costimulatory molecule ICOS may play a more indispensable role in ILC3 biology than in T helper cell or ILC2 processes.

### ICOS^+^ ILC3s show more activation-associated and helper-like characteristics

**Fig. 2:**
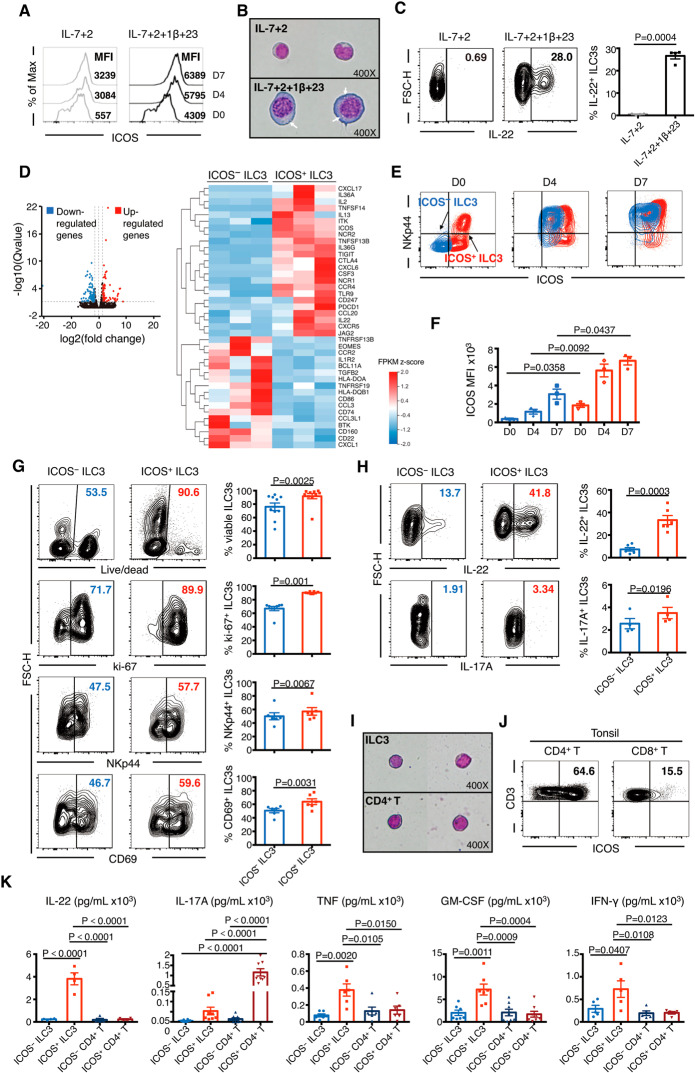
ICOS^+^ ILC3s are more like helper cells than ICOS^−^ ILC3s based on their transcription profile and functions. **A**–**C** Purified ILC3s from the same donor were stimulated by interleukin (IL)-2 + IL-7 or IL-2 + IL-7 + IL-1β + IL-23. **A** Median fluorescence intensity (MFI) for ICOS on ILC3s. **B** Morphological comparison of ILC3s (white arrows indicate vesicles) on Day 7. **C** Representative flow plots and quantification of IL-22^+^ ILC3s (*n* = 4). **D** Volcano plot comparison (left) of differentially expressed genes (DEGs) between tonsillar ICOS^−^ and ICOS^+^ ILC3s. The heatmap (right) shows the DEGs related to the immune system and environmental information processing. DEGseq analysis (log 2 of fold change ≥ 1.5, *Q* ≤ 0.005) was used to identify the DEGs (*n* = 3). The results are shown as a scatter plot of the Z score (row direction) for fragments per kilobase million mapped (FPKM). **E**–**H** Purified tonsillar ICOS^−^ and ICOS^+^ ILC3s were treated with IL-7 + IL-2 + IL-1β + IL-23 and analyzed on Day 4 and/or 7. **E** Representative fluorescence-activated cell sorting (FACS) profiles for ICOS^−^ ILC3s (light blue) and ICOS^+^ ILC3s (light red) overlapped by ICOS and NKp44 expression at Day 0, 4, and 7. **F** Change in relative ICOS expression on ICOS^−^ and ICOS^+^ ILC3s on Day 0, 4, and 7 (*n* = 3). **G**, **H** Flow cytometry profiles and summary of the frequencies of viable (G upper), ki-67^+^ ILC3s (G upper-middle), NKp44^+^ ILC3s (G lower-middle), and CD69^+^ ILC3s (G lower) as well as IL-22^+^ ILC3s (H upper) and IL-17A^+^ ILC3s (H lower) treated with IL-7 + IL-2 + IL-1β + IL-23 on Day 4 (*n* = 4–10). **I** Morphological comparison of ILC3s vs. CD4^+^ T cells sorted from tonsillar lymphocytes. **J** Representative FACS profiles for ICOS expression of tonsillar CD4^+^ (left) and CD8^+^ T cells (right). **K** Sorted tonsillar ICOS^−/+^ ILC3s and ICOS^−/+^ CD4^+^ T cells were incubated at the same cell concentration and stimulated by IL-7 + IL-2 + IL-1β + IL-23 (for ILC3s) or anti-CD3/CD28 antibody (for CD4^+^ T cells) in vitro. On Day 4, IL-22, IL-17A, total TNF, GM-CSF, and IFN-γ levels in the supernatants were determined using enzyme-linked immunosorbent assay (ELISA) and cytometric bead array (CBA) (**J**). The results generated from different donors (*n* = 4–8) were combined. The error bars indicate the mean ± SEM. Statistical significance was determined using Student’s paired *t-*test (**C**, **F**–**H**) or unmatched one-way analysis of variance (ANOVA) and Tukey’s multiple comparison test (**K**)

To investigate the effect of ICOS on the characteristics of ILC3s, tonsillar ILC3s, characterized by RORγt (Supplementary Fig. 2a, b), were isolated and then activated in vitro. When stimulated by IL-7 + IL-2 + IL-1β + IL-23, the ICOS level on ILC3s further increased, and ILC3s were completely converted to ICOS^+^ ILC3s on Day 4. In contrast, despite some increase in expression, the degree of ICOS upregulation was not as remarkable after IL-7 + IL-2 stimulation as that after IL-7 + IL-2 + IL-1β + IL-23 stimulation (Fig. 2A). This result was confirmed using PB-derived ILC3s (Supplementary Fig. 2c). In line with this, ILC3s were enlarged after IL-7 + IL-2 + IL-1β + IL-23 stimulation, with inflated nuclei and cytoplasm and several vesicles within the cytoplasm (Fig. 2B), which were accompanied by enhanced ILC3 survival, activation, and cytokine (IL-22, IL-17A, TNF, IFN-γ, and GM-CSF) production (Fig. 2C, Supplementary Fig. 2d, e). These findings suggested that ICOS may act as an activation-associated molecule on the ILC3 surface.

To further elucidate the role of ICOS in ILC3s, tonsillar ILC3s were divided into ICOS^−^ and ICOS^+^ ILC3s and sorted for RNA sequencing analysis (Fig. 2D, Supplementary Fig. 3a, b, Supplementary Table 1). Interestingly, ICOS^+^ ILC3s shared some genes with activated ILCs (*NCR1, NCR2, CD160*, and *IL13*) [20, 21] and T helper cells (*IL2, IL22, CSF3, TIGIT, CTLA4, PDCD1, TNFSF14*, and *TNFSF13B*). However, ICOS^−^ ILC3s also had the expression of some genes involved in antigen presentation, such as the MHCII family genes [22] (*HLA-DQB1, HLA-DOA*, and *CD74*) and B-cell-associated genes (*CD86, BTK, CD22, TNFRSF13B*, and *TNFRSF19*) [23]. Next, ICOS^−^ and ICOS^+^ ILC3s were stimulated by IL-7 + IL-2 + IL-1β + IL-23 in vitro. Although ICOS expression increased on ICOS^−^ and ICOS^+^ ILC3s, the ICOS levels on ICOS^+^ ILC3s were higher than those on autologous ICOS^−^ ILC3s and peaked on Day 4 after activation (Fig. 2E, F). In line with the increased ICOS expression, the proportions of viable ILC3s, ki-67^+^ ILC3s, NKp44^+^ ILC3s, and CD69^+^ ILC3s in ICOS^+^ ILC3s were higher than those in ICOS^−^ ILC3s, and the same trend was observed for intracellular IL-22 and IL-17A expression (Fig. 2G, H). These observations indicated that ICOS might act as an activation-associated molecule rather than a specific biomarker of ILC3s.

In line with the ICOS expression results, ILC3s and CD4^+^ T cells showed remarkably similar cell size and morphology and a similar nuclear-cytoplasmic ratio, with oval- or bean-shaped single nuclei and oligoplasmic lymphoid morphology (Fig. 2I, J). To investigate their functional heterogeneity, ICOS^−/+^ ILC3s and ICOS^−/+^ CD4^+^ T cells were cultured at the same cell count and concentration. The results showed that the levels of cytokines secreted by ICOS^+^ ILC3s, especially that of IL-22, were higher than those secreted by ICOS^−^ ILC3s at Day 4 (Fig. 2K) and Day 7 (Supplementary Fig. 4a). The levels of some cytokines, such as IL-22, TNF, IFN-γ, and GM-CSF, produced by ICOS^+^ ILC3s were higher than those produced by ICOS^+^ CD4^+^ T cells. In contrast, the IL-17A-secreting capacity of ICOS^+^ ILC3s was lower than that of ICOS^+^ CD4^+^ T cells. These results indicated that tonsil-resident ICOS^+^ ILC3s might play a unique role in the immune environment via specific secretion of cytokines even though there are fewer ICOS^+^ ILC3s than T helper cells.

ICOS costimulation promotes the production of IL-10 by regulatory T cells (Tregs) [8], and ILCs contain a subgroup of IL-10^+^ ILCs, which are known as regulatory ILCs (ILCregs) [24]. Thus, we investigated whether ICOS^+^ ILC3s have the characteristics of ILCregs. Neither ICOS^−^ ILC3s nor ICOS^+^ ILC3s had the ability to secrete IL-10; however, both ICOS^-^ CD4^+^ T and ICOS^+^ CD4^+^ T cells secreted some IL-10 (Supplementary Fig. 4b), in agreement with previous studies [25, 26], suggesting that ICOS^+^ ILC3s and ILCregs [24] are two distinct and nonoverlapping ILC subpopulations. Collectively, the role of ICOS in ILC3s is heterogeneous, distinct from that in ICOS^+^ T helper cells, and cell type-specific.

### ICOS costimulation enhances ILC3 functions

**Fig. 3:**
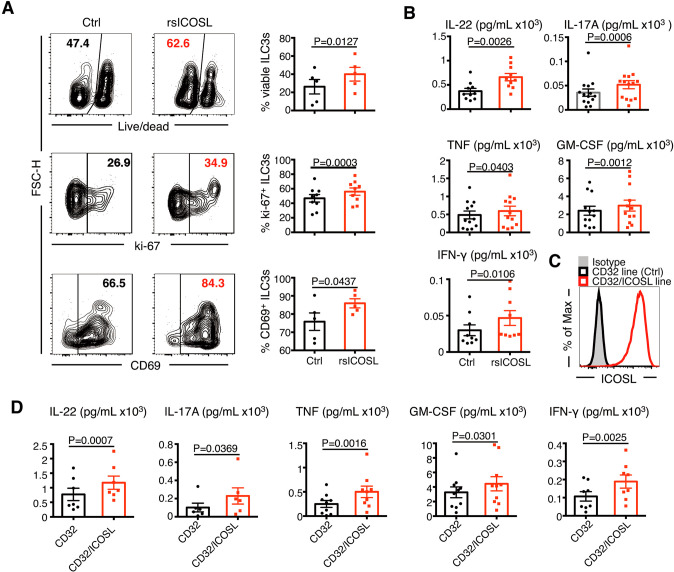
ICOS costimulation enhances the survival, proliferation, and activation of ILC3s. **A**, **B** Tonsillar ILC3s were incubated in human recombinant soluble ICOSL protein (rsICOSL)-precoated or uncoated wells (control) in the presence of IL-7, IL-1β, and IL-23 for 4 days. **A** Representative flow plots and quantification of viable ILC3s (upper), ki-67^+^ ILC3s (middle), and CD69^+^ ILC3s (lower). The numbers in the quadrant represent the percentages of cells (*n* = 5–9). **B** Cytokines in the supernatants at Day 4 as determined using ELISA and CBA (*n* = 9–14). **C** Histogram comparing ICOSL expression among the CD32/ICOSL-expressing cell line (red line) and CD32-expressing cell line (black line), with the isotype control shown in gray. **D** Purified tonsillar ILC3s were cocultured with the parental CD32 line (control) and CD32/ICOSL line in the presence of IL-7, IL-1β, and IL-23 for 4 days. Cytokine levels in the supernatants were determined using ELISA and CBA (*n* = 6–10) on Day 4. The error bars indicate the mean ± SEM, and *P* values were determined using a two-tailed Student’s paired *t-*test

To elucidate the influence of the ICOS/ICOSL interaction on human ILC3s, we used ICOS costimulation assays as previously reported [27, 28]. The results showed that costimulation of ILC3s with precoated human recombinant soluble ICOSL protein (rsICOSL) enhanced the survival, proliferation, and activation of ILC3s, as indicated by an increased proportion of viable, ki-67^+^ and CD69^+^ ILC3s (Fig. 3A), even without NKp44^+^ and CD25^+^ ILC3 expansion (Supplementary Fig. 5a). In addition, the concentrations of IL-22, IL-17A, IFN-γ, TNF, and GM-CSF were significantly upregulated after ICOSL engagement (Fig. 3B). However, free rsICOSL failed to enhance cytokine production by ILC3s (Supplementary Fig. 5b), which indicated that the effect of ICOS costimulation on ILC3s primarily depended on ILC3 cell-to-cell contact via surface molecules with the ICOSL provider rather than via interaction with free ICOSL in the microenvironment.

To verify the immunological function of ICOSL^+^ cells on ILC3s, a human cell line expressing full-length ICOSL (CD32/ICOSL-expressing cells) was generated as previously described [26] (Fig. 3C) and cocultured with tonsillar ILC3s. Consistent with the effect of precoated rsICOSL Fig. 3B, the CD32/ICOSL-expressing cell line also increased the production of IL-22, IL-17A, IFN-γ, TNF, and GM-CSF by ILC3s (Fig. 3D, Supplementary Fig. 5c). Collectively, the ICOS/ICOSL pathway provides crucial costimulatory signals that reinforce ILC3 activation, and ILC3s are likely to receive activation-modulating signals by interacting with ICOSL^+^ cells in the immune microenvironment.

### Bidirectional promotion between tonsillar ILC3s and autologous B cells

**Fig. 4:**
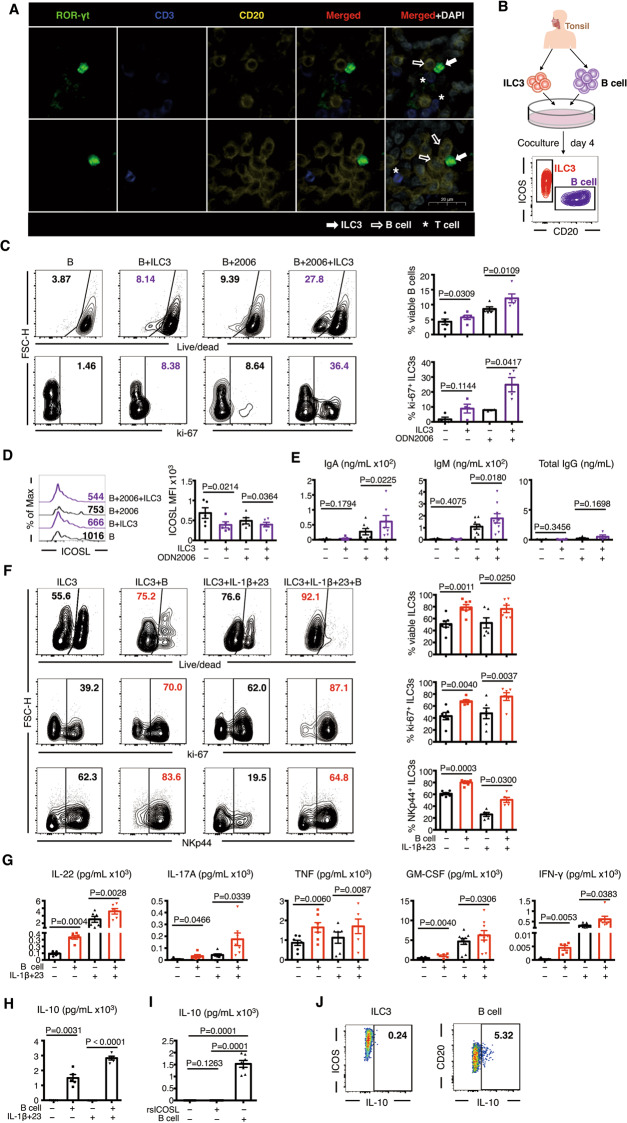
ILC3s and B cells in coculture promote the specific functions of each other. **A** Immunofluorescence-based histological analysis of tonsils. ILC3s (ROR-γt^+^ CD3^−^) and B cells (CD20^−^) were in close contact with each other in vivo. Solid arrows, ILC3s; open arrows, B cells; *, T cells. Scale bar = 20 μm. **B**–**I** Tonsillar ILC3s and B cells (CD3^−^ CD19^+^) sorted from the same donor were cocultured for 4 days and subjected to FACS analysis (**B**), and ELISA and CBA were used to determine the phenotypes and functions of ILC3s (CD45^+^ CD19^−^ CD20^−^ ICOS^+^) and B cells (CD45^+^ CD19^−/+^ CD20^+^ ICOS^−^). **C**–**E** B cells were cocultured with autologous ILC3s at a 5:1 ratio in the presence or absence of ODN2006 for 4 days. **C** Representative flow plots and quantification of viable (upper) and ki-67^+^ (lower) B cells (*n* = 4–5). **D** MFI and quantification of ICOSL on B cells incubated for 4 days with or without ILC3s (*n* = 5). **E** ELISA and CBA for IgA, IgM, and total IgG in the supernatants of B cells incubated for 4 days with or without ILC3s (*n* = 4–9). **F**–**I** ILC3s were cocultured with autologous B cells at a 1:15 ratio in the presence of IL-7 + IL-2 or IL-7 + IL-2 + IL-1β + IL-23 for 4 days. **F** Representative flow plots and quantification of viable (upper), ki-67^+^ (middle), and NKp44^+^ ILC3s (down) (*n* = 5–7). **G** ELISA and CBA for IL-22, IL-17A, total TNF, GM-CSF, and IFN-γ in the supernatants of ILC3s incubated for 4 days with or without B cells (*n* = 5–9). **H**, **I** ELISA for IL-10 in the supernatant of ILC3s incubated for 4 days with or without B cells (H) and ICOSL protein (**I**). The results are representative of five (**H**) or seven (**I**) experiments. The error bars indicate the mean ± SEM. **J** Representative flow plots of IL-10^+^ ILC3s and IL-10^+^ B cells after coculture for 4 days. Statistical significance was determined using Student’s paired *t-*test (**C**–**H**) or matched one-way ANOVA and Tukey’s multiple comparison test (**I**)

B cells are not only the main provider of ICOSL [9, 29] but also the predominant immune cells in the tonsil (Supplementary Fig. 6a). Resembling T cells, RORγt^+^ CD3^−^ ILC3s also colocalized with B cells in vivo (Fig. 4A). To study the bidirectional effects between ILC3s and B cells, autologous tonsillar ILC3s and B cells were sorted and cocultured at different ratios (Fig. 4B). The results showed that ILC3s and B cells promoted the survival of each other, and the survival of these cells was ratio-dependent (Supplementary Fig. 5b).

Regarding the influence of ILC3s on B cells, ILC3s promoted the survival and proliferation of B cells, particularly in the presence of oligonucleotide (ODN) 2006 (a class B CpG ODN that can be used for B-cell activation as a ligand of TLR9, Fig. 4C), and the level of detectable ICOSL on the surface of B cells was reduced (Fig. 4D), suggesting that ICOSL was shed due to interactions with ICOS^+^ ILC3s [30]. Because IL-1β induces the production of IgA, IgM, and IgG by B cells (Supplementary Fig. 7a), in this ILC3-B-cell coculture system, IL-1β was excluded. Functionally, ILC3s promoted the secretion of IgA and IgM, but not IgG, by B cells; however, this impact was not remarkable in the absence of ODN2006 stimulation (Fig. 4E). As the ICOSL level is downregulated after interaction with ICOS [31], the ICOS signaling pathway may participate in the process of B-cell priming by ILC3s.

Regarding the effect of B cells on ILC3s, B cells were cocultured with ILC3s in the presence of IL-7 + IL-2 or IL-7 + IL-2 + IL-1β + IL-23 stimuli. We found that after coculture with B cells, regardless of the addition of IL-1β and IL-23, the proportion of viable ILC3s, ki-67^+^ ILC3s, and NKp44^+^ ILC3s as well as the number of ILC3s increased (Fig. 4F, Supplementary Fig. 7b). CD69 expression on ILC3s was not affected after coculture with B cells (Supplementary Fig. 7c). Moreover, B cells promoted ILC3 production of cytokines, including IL-22, IL-17A, IFN-γ, TNF, and GM-CSF (Fig. 4G, Supplementary Fig. 7d). In the ILC3-B-cell coculture system, ODN2006 was added for B-cell activation; however, despite ICOS^+^ ILC3s showing expression of TLR9 transcripts (Fig. 2D), ODN2006 did not significantly influence ILC3 activation (Supplementary Fig. 7e, f).

In the supernatant of the ILC3 and B-cell coculture system, we found high IL-10 levels (Fig. 4H), which was consistent with previous findings that ILC3s induce B-cell differentiation into IL-10-producing regulatory B cells (Bregs) [4]. We next determined whether IL-10 was generated by ILC3s. We found that IL-10 was undetectable in the supernatant of ILC3s regardless of stimulation by IL-7 + IL-2 + IL-1β + IL-23 or rsICOSL, but its expression was triggered when B cells were added. (Fig. 4H, I). Furthermore, the IL-10 in the ILC3-B-cell coculture system was mainly derived from B cells and not ILC3s (Fig. 4J). These results suggest that neither activation by IL-7 + IL-2 + IL-1β + IL-23 nor costimulation of ICOS triggers IL-10 production by ILC3s.

### Reciprocal promotion of ILC3s and B cells partially requires the ICOS/ICOSL interaction

**Fig. 5:**
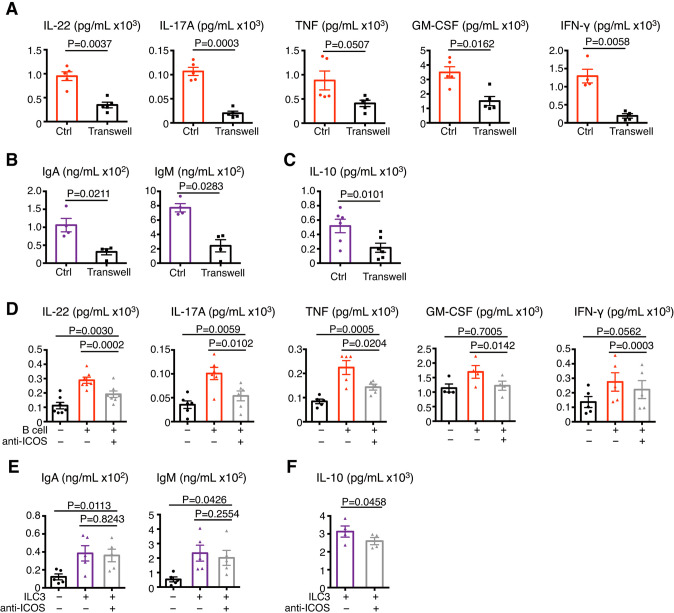
The bidirectional promotion of ILC3s and B cells partially requires ICOS/ICOSL-induced cell‒cell contact. **A**–**C** Blocked of tonsillar ILC3-B-cell cell-cell contact in transwell cocultures. ELISA and CBA for IL-22, IL-17A, total TNF, GM-CSF, IFN-γ (**A**), IgA, IgM (**B**), and IL-10 (**C**) in the supernatant of ILC3s and B cells cocultured at a 1:5 ratio for 7 days in the presence of IL-7, IL-23, and ODN2006 with or without a transwell (ILC3s in the upper well, *n* = 4–6). **D**–**F** Effects of ICOS blockade on the ILC3-B-cell interaction. **D** ELISA and CBA for IL-22, IL-17A, total TNF, GM-CSF, and IFN-γ in the supernatants of ILC3s only (black), cocultured ILC3s and B cells (red), and cocultured ILC3s and B cells with an anti-ICOS antibody (gray) incubated at a 1:15 ratio for 4 days in the presence of IL-7, IL-23, and ODN2006 (*n* = 5–7). ELISA for IgA, IgM (**E**), and IL-10 (**F**) in the supernatants of B cells only (black), cocultured ILC3s and B cells (purple), and cocultured ILC3s and B cells with anti-ICOS antibody (gray) incubated at a 1:5 (**E**) or 1:15 (**F**) ratio for 4 days in the presence of IL-7, IL-23, and ODN2006 (*n* = 4–5). The error bars indicate the mean ± SEM, and statistical significance was determined using Student’s paired *t*-test (**A**–**C**, and **F**) or matched one-way ANOVA and Tukey’s multiple comparison test (**D**, **E**)

We next explored whether the reciprocal effects of ILC3s and B cells are contact-dependent or contact-independent. A Transwell assay showed that when cell contact was blocked, the B-cell-induced ILC3-specific production of IL-22, IL-17A, IFN-γ, TNF, and GM-CSF was eliminated (Fig. 5A). Similarly, the ILC3-induced upregulation of IgA and IgM secretion by B cells was inhibited (Fig. 5B). These observations were in line with the decreased IL-10 levels in B cells (Fig. 5C). Although some secreted extracellular factors, such as ILC3-derived BAFF and B-cell-derived IL-15, promote crosstalk between ILC3s and B cells [4, 5], these results indicate that direct contact between ILC3s and B cells is essential for their reciprocal promotion.

To further investigate the role of ICOS signaling in the ILC3-B-cell interaction, we conducted blocking assays using an ICOS-neutralizing antibody followed by functional tests of ILC3s and B cells. ICOS blockade resulted in a partial decrease in IL-22, IL-17A, TNF, GM-CSF, and IFN-γ secretion by ILC3s (Fig. 5D) but little reduction in IgA, IgM, and IL-10 expression in B cells (Fig. 5E, F). These results suggest that ICOS is partially involved in promoting ILC3 activation during the interaction of ILC3s and B cells. Although IL-21 is involved in ICOS-mediated Tfh and B-cell interactions in lymphoid tissues, such as the tonsil [32, 33], neither ICOS^-^ ILC3s nor ICOS^+^ ILC3s produced IL-21 (Supplementary Fig. 7g). Collectively, the ILC3-B-cell interaction is partially ICOS signaling-dependent, and it may involve other molecules that induce contact between ILC3s and B cells.

### B-cell-reinforced ILC3 activation requires the cooperation of ICOS and CD40 signaling

**Fig. 6:**
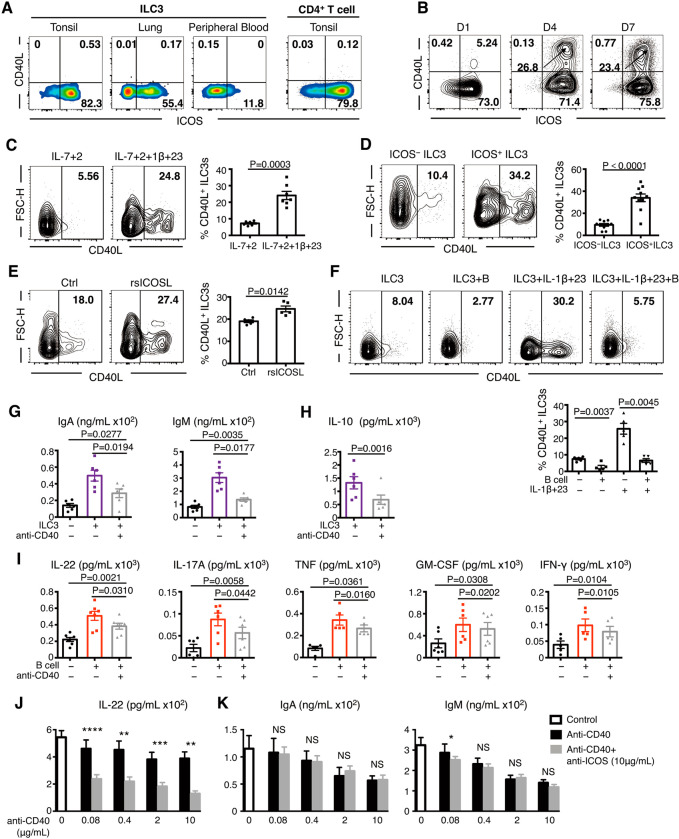
B-cell-induced activation of ILC3s requires the cooperation of ICOS and CD40 signaling. **A** Representative flow plots for CD40L and ICOS expression in ILC3s from human tonsil, distal and normal lung samples from donor with lung cancer, PB of healthy donor, and CD4^+^ T cells from human tonsil. **B**, **C** Representative flow plots and quantification of CD40L^+^ ILC3s in tonsillar ILC3s stimulated by IL-2 + IL-7 + IL-1β + IL-23 for 1, 4, and 7 days (**B**) or incubated with or without IL-1β + IL-23 for 4 days (**C**). The results are representative of three donors in (**B**) and seven experiments in (**C**). **D** Representative flow plots and quantification of CD40L^+^ ILC3s in ICOS^−^ and ICOS^+^ ILC3s stimulated by IL-2 + IL-7 + IL-1β + IL-23 for 4 days (*n* = 10). **E** Representative flow plots and quantification of CD40L^+^ ILC3s in purified tonsillar ILC3s incubated with or without ICOSL protein in the presence of IL-7, IL-1β, and IL-23 for 4 days (*n* = 5). **F** Representative flow plots and quantification of CD40L^+^ ILC3s in tonsillar ILC3s cocultured with autologous B cells at a 1:15 ratio in the presence of IL-7 + IL-2 or IL-7 + IL-2 + IL-1β + IL-23 for 4 days (*n* = 5). The effects of CD40 blockade on the ILC3-B-cell interaction were assessed (**G**–**I**). ELISA of IgA, IgM (**G**), and IL-10 (**I**) in the supernatants of B cells only (black), cocultured ILC3s and B cells (purple), and cocultured ILC3s and B cells with anti-CD40 antibody (gray) incubated at 1:5 (**E**) or 1:15 (**F**) ratios for 4 days in the presence of IL-7, IL-23, and ODN2006 (*n* = 6). **I** ELISA and CBA for IL-22, IL-17A, total TNF, GM-CSF, and IFN-γ in the supernatants of ILC3s only (black), cocultured ILC3s and B cells (red), and cocultured ILC3s and B cells with anti-CD40 antibody (gray) incubated at a 1:15 ratio for 4 days in the presence of IL-7, IL-23, and ODN2006 (*n* = 5–7). **J**, **K** Tonsillar ILC3s were cocultured with autologous B cells in the absence (blank column) or presence of a different concentrations of anti-CD40 antibody (black column) plus the ideal concentration of anti-ICOS antibody (gray column) for 4 days. **J** ELISA of IL-22 in the supernatant of the ILC3-B-cell coculture system (ratio = 1:15). **K** CBA of IgA and IgM in the supernatant of the ILC3 and B-cell coculture system (1:5). The results are representative of five (**J**) or six (**K**) experiments. The error bars indicate the mean ± SEM. *P < 0.05, **P < 0.005, ***P < 0.0005, ****P < 0.0001, and NS *P* ≥ 0.05. Statistical significance was determined using Student’s paired *t*-test (**C**–**F**, **H**, **J,** and **K**) or matched one-way ANOVA and Tukey’s multiple comparison test (**G**, **I**)

The costimulatory molecule CD40 generally supports the ICOS/ICOSL interaction in T-cell-B-cell responses [9, 34]. Thus, we investigated the role of CD40 signaling in the ILC3-B-cell interaction. Of note, CD40L expression was not detected on fresh tonsil-derived, lung-derived, or PB-derived ILC3s (Fig. 6A) but appeared on the ILC3 surface after activation by IL-7 + IL-2 + IL-1β + IL-23 (Fig. 6B). The percentage of induced CD40L^+^ ILC3s was further increased after activation (Fig. 6C). Moreover, ICOS^+^ ILC3s had a stronger potential for CD40L expression, and the percentage of CD40L^+^ ILC3s increased after ICOSL engagement (Fig. 6D, E). During Tfh-B-cell responses, the ICOS/ICOSL interaction promotes the expression of CD40L on T cells [9, 34, 35], suggesting that ILC3s may function similarly to Tfh cells during interaction with B cells, and the effect of CD40 likely occurs later than the ICOS/ICOSL interaction.

To determine the role of CD40 signaling in the ILC3-B-cell interaction, we used a CD40-neutralizing antibody in the ILC3 and B-cell coculture system to perform a CD40 blockade assay. The results showed that once ILC3s interacted with B cells, CD40L on ILC3s was undetectable (Fig. 6F), and CD40 blockade inhibited the ILC3-induced upregulation of IgA, IgM, and IL-10 levels in B cells (Fig. 6G, H). In addition, the B-cell-induced increase in ILC3 cytokine secretion was partially inhibited after CD40 blockade (Fig. 6I), suggesting that CD40 signaling mediated the interaction between the two cells.

To further investigate the relationship between ICOS and CD40 signaling in the ILC3-B-cell interaction, we prepared anti-ICOS and anti-CD40 antibodies in gradient concentrations. Regarding ILC3s, the inhibitory effect of anti-CD40 antibody on ILC3-produced IL-22 was dose-dependent in the absence of anti-ICOS antibody. In the presence of an optimal dose of anti-ICOS antibody plus an altered concentration of the anti-CD40 antibody, IL-22 was inhibited much more significantly; however, the dose-dependence of the anti-CD40 antibody was partially weakened (Fig. 6J). In B cells, IgA and IgM expression was inhibited after CD40 blockade in a dose-dependent manner in the absence of anti-ICOS antibody. The anti-ICOS antibody acted synergistically with the anti-CD40 antibody; however, the inhibitory function of the anti-ICOS antibody was concealed in the presence of abundant anti-CD40 antibody (Fig. 6K). In summary, ICOS and CD40 signaling play a synergistic role in the B-cell-mediated promotion of ILC3s, with ICOS signaling acting as a major component. In addition, ILC3-induced enhancement of B-cell functions is primarily dependent on CD40 signaling.

## Discussion

ILC3s are equipped with not only APC-like features such as MHCII [16, 22, 23, 36] and OX40L [37, 38] expression but also helper-like characteristics such as expression of the costimulatory molecule CD40L [9, 34, 35], and thus, they participate in T-cell and APC regulation. The complex functions of ILC3s in the immune microenvironment are determined by their phenotype; however, the underlying mechanism is not completely understood, especially in humans. Our findings identified the expression of ICOS on ILC3s at the protein level in multiple human tissues. Although ICOS expression can also be found on ILC2s [11, 39] and ILC1s, we failed to compare the ICOS-related differences among human ILC subsets because of the low absolute number of ILCs in the majority of tissues.

ICOS^+^ ILC3s share many features with activated T helper cells, such as the expression of IL-2, IL-22, NKp44, NKp46, CXCR5, and PD-1; however, their features are not entirely the same [40]. For instance, CD28, a secondary signal involved in T-cell activation, commonly colocalizes with ICOS on T helper cells, but it is almost undetectable on solid tissue- or PB-derived ILC3s. In addition, RNA sequencing analysis revealed the expression of some costimulatory molecules in ICOS^+^ ILC3s, such as LIGHT, TIGIT, and CTLA4 (Fig. 2D), so additional studies are required. The increased ICOS level in ILC3s was accompanied by enhanced survival and proliferation and specific cytokine production after IL-7 + IL-2 + IL-1β + IL-23 activation, indicating that ICOS can be considered an activation-related molecule for ILC3s. Although the ICOS^−^ and ICOS^+^ ILC3 profiles differed, they had similar phenotypes after stimulation with IL-7 + IL-2 + IL-1β + IL-23. In ICOS costimulation assays for ILC3s, suboptimal stimuli were identified as indispensable, similar to the case for ICOS costimulation of T cells [27, 28]. This result suggests that ICOS signaling may be a secondary signal for ILC3 activation, assuming that IL-1β and IL-23 act as the primary activators.

B cells can acquire help from Th cells and ILC3s. In this study, the T-cell-independent B-cell help of ILC3s resembled the interaction of Tfh and B cells and required ICOS and CD40 signaling; however, the difference was that the B-cell-trophic cytokine IL-21 [32, 33], which is related to Tfh cells, was undetectable in ICOS^−^ and ICOS^+^ ILC3s. Further study is required to determine whether there is a competitive relationship between T-cell-dependent and ILC3 (helper ILC3 and LTi)-dependent B-cell switching and to identify any related factors [2]. In contrast to the promoting effects of ILC3s on B cells in the human spleen [5], we found that tonsillar ILC3s promoted the secretion of IgA and IgM but not IgG, indicating that in addition secreting cytokines such as IL-22, ILC3s also regulate mucosal immunity by promoting IgA and IgM production by B cells.

To date, there have been few studies regarding the influence of other immune cells on ILC3s and the relevant mechanisms. Despite the low cell number and proportion in human tissues, we primarily focused on the effects of B cells on ILC3s during their interaction. Of note, in the absence of oppression by T cells [41], B cells promoted ILC3 survival, proliferation, and function, which was dependent on ICOS and CD40 signaling. In addition to B cells, ICOSL-expressing cells include DCs [42], macrophages [39], and somatic cells, such as human umbilical vein endothelial cells [43], and even ILC2s [11, 39, 44]. Our finding that ICOSL was undetectable on human tonsillar ILC3s contradicted the findings of ICOSL expression on murine ILC2s [11], thereby providing evidence for a difference between ILC2s and ILC3s [11, 45]. Thus, the ICOS-mediated interactions between ILC3s and other cell types need further study. Considering that IL-1β and IL-23 are triggered by foreign antigens [38, 41], together with the fact that the ICOS/ICOSL pathway mediates the inflammatory response [46, 47], the role of the ICOS/ICOSL pathway in the interaction between ILC3s and B cells in diseases like infection and tumorigenesis needs to be further studied.

ILC3s maintain a strong capacity for cytokine production even after prolonged stimulation in vitro, and ILC3s show obviously high levels of proliferation and activation once they are isolated from T cells. However, in human and murine tissues, ILC3s represent a small population [13, 48, 49] in both steady state [13, 50] and disease [13, 51]. Thus, it is possible that non-tissue-specific and broad-spectrum inhibitory factors around ILCs restrain the expansion of ILCs inside the body. These factors may have competitive effects against prominent T cells [41] or suppressive effects against immune regulatory cells, such as Tregs, Bregs, and ILCregs, [24] and may include cytokines that maintain immune homeostasis among multiple immune cells.

In conclusion, ICOS expression on human ILC3s functions as a secondary activation signal and contributes to ILC3 survival, proliferation, and production of cytokines, including IL-22 and IL-17A. The direct contact between autologous ILC3s and B cells induced by ICOS and ICOSL interaction facilitates the activation-associated state of ILC3s, which is synergistically mediated by CD40 signaling. ILC3 help in B-cell Ig class switching and differentiation primarily requires CD40 signaling and, to a lesser extent, ICOS signaling (Supplementary Fig. 8). These observations indicate the unique and nonredundant role of ILC3s in the immune microenvironment and their significant implications for studies investigating the T-cell-independent B-cell response during lymphoid tissue formation and Ig class switching-associated diseases, such as common variable immunodeficiency.

## Methods

### Human sample and tissue processing

Tonsil tissue samples were obtained from pediatric patients undergoing routine tonsillectomy at the First Hospital of Jilin University. Human distal lung biopsies (more than 5 cm from the tumor lesion) were obtained from lung cancer patients at the Second Hospital of Jilin University. Peripheral blood (PB) samples from healthy donors were obtained from the Blood Bank Center of Jilin Province, Changchun, China. All study participants provided informed written consent (Ethics Committee of the First Hospital of Jilin University, Clinical Trials and Research Approval No. 2020-426).

Fresh tonsil and lung tissues were mechanically dissociated on ice in RPMI-1640 containing 2% fetal bovine serum (FBS, Gibco, New Zealand) and then digested for 30–45 min at 37 °C with 500 μg/mL type IV collagenase (Gibco, New York) and 5 μg/mL DNase I (Sigma‒Aldrich, Shanghai) in culture medium. Subsequently, the cells were washed with PBS + 2% FBS and passed through a 70 μm filter to generate a single-cell suspension. Before antibody staining for cell sorting, tonsillar and PB mononuclear cells were isolated using Ficoll-Paque Plus medium (GE Healthcare, Uppsala) through density gradient centrifugation.

### Flow cytometry and cell sorting

Single-cell preparations were stained with antibodies as shown in Supplementary Table 2. According to the expression of multiple biomarkers, ILC1s (CD45^+^ CD3^−^ Lineage^−^ (CD3 CD19 CD20 CD14 CD94 CD34 CD1a CD11c CD123 TCRα/β TCRγ/δ FcεRIα) CD127^+^ CRTH2^−^ CD117^−^), ILC2s (CD45^+^ CD3^−^ Lineage^−^ CD127^+^ CRTH2^+^), ILC3s (CD45^+^ CD3^−^ Lineage^−^ CD127^+^ CRTH2^−^ CD117^+^), CD4^+^ T cells (CD45^+^ CD3^+^ CD4^+^), CD8^+^ T cells (CD45^+^ CD3^+^ CD4^−^) and B cells (CD45^+^ CD3^−^ CD19^+^) were defined and then subjected to surface, intracellular, and intranuclear staining to investigate phenotypes and cytokine production. Dead cells were excluded from analysis using the LIVE/DEAD Fixable Aqua Dead Cell Stain (Invitrogen, Oregon). For intranuclear staining, the Transcription Factor Fixation/Permeabilization Buffer Set (BioLegend, San Diego) was used, and cells were stained with anti-ki-67 (clone ki-67; eBioscience BioLegend, San Diego), anti-RORγt (clone Q21-559; BD Biosciences, San Jose), anti-T-bet (clone 4B10; BD Biosciences, San Jose), and anti-GATA3 (clone 16E10A23; BioLegend, San Diego) antibodies. For cytokine detection, Fixation and Permeabilization Solution (BD Biosciences, San Jose) was used, and cells were stained with anti-IL-17A (BL168; BioLegend, San Diego) and anti-IL-22 (clone 22URTI; eBioscience, San Diego) antibodies. Data were analyzed with FlowJo^TM^ 10.0.3 software.

Before sorting using flow cytometry, tonsillar and PB mononuclear cells were depleted of T cells and B cells. Briefly, cells were labeled with biotin-conjugated anti-CD3 and anti-CD19 antibodies (BioLegend, San Diego) followed by incubation with anti-biotin microbeads (Miltenyi, Germany). Cells were then passed through an LS column (Miltenyi) using a MultiMACS Separator (Miltenyi) according to the manufacturer’s instructions. Subsequently, the ILC subsets from the microbead-unlabeled mononuclear cells were isolated using FACSAria (BD Biosciences, San Jose). The staining and gating were performed as described above. In addition, ICOS^+^ T cells (CD3^+^ CD4^+^ CD19^−^) and B cells (CD3^−^ CD19^+^) were isolated with a FACSAria from microbead-labeled mononuclear cells. All cells utilized for experiments had a purity of higher than 95%.

### ICOSL-expressing cell lines

Human ICOSL-expressing cells were generated using retroviral-mediated transduction. Briefly, using the pLent-EF1a-FH-CMV-copGFP-P2A-Puro lentivirus vector, the full-length coding sequence for human FC-GAMMARIIC (GenBank: U90939.1) plus human ICOSLG (NM_015259) or FC-GAMMARIIC alone was transfected into K562 cell lines. Subsequently, puromycin dihydrochloride (Santa Cruz Biotechnology, Dallas) was used to obtain CD32/ICOSL and CD32 control lines with a purity higher than 95%. The cell lines were prepared by Shandong Weizhen Biotechnology Co., Ltd. The cells were used in the ICOS costimulation of ILC3s. The cells were cultured in DMEM containing 10% FBS, and 2 µg/mL puromycin was added to maintain cell purity.

### Cell culture and ICOS costimulation

Sorted tonsillar and PB ILC3s were incubated in RPMI-1640 containing 10% FBS and 1% penicillin/streptomycin in the presence of 50 ng/mL IL-7 (PeproTech, Cranbury) and 100 U/mL IL-2 (PeproTech, Cranbury) in 96-well U type- or flat-bottom culture plates at a concentration of 2×10^4^ cells/well. For ILC3 activation, 10 ng/mL IL-1β and 50 ng/mL IL-23 were added to the medium for 4 or 7 days. For ICOS costimulation, ILC3s were incubated with precoated or free recombinant human B7-H2 protein (R&D Systems, Minneapolis) in the presence of 50 ng/mL IL-7, 10 ng/mL IL-1β and 50 ng/mL IL-23 for 4 days. In addition, ILC3s were cocultured with the CD32/ICOSL-expressing cell line or parental CD32-expressing cell line (control) at a ratio of 4:1 for 4 days. In the ILC3 and B-cell coculture assay, autologous tonsillar ILC3s and B cells were coincubated (ILC3/B-cell ratio of 1:15 or 1:5) in flat-bottom culture plates for 4 days in the presence of suboptimal ILC3 stimuli (50 ng/mL IL-7 plus 50 ng/mL IL-23) and B-cell stimuli (1 μM ODN2006, InvivoGen, San Diego). In the ICOS and CD40 blockade assays, before coculturing, ILC3s and B cells were incubated with 1 μg/mL anti-ICOS (AnCell, Bayport, clone ANC6C6) and 1 μg/mL anti-CD40 (BioLegend, San Diego, clone W17212H) antibodies, respectively, at 37 °C for 2 h, and autologous ILC3s and B cells were then cocultured as described above for 4 days. In some experiments, CD4^+^ ICOS^−^ and CD4^+^ ICOS^+^ T cells were sorted from tonsils and cultured under the same ILC3 activation conditions in the presence of Dynabeads human T-Activator CD3/CD28 (T-cell/Dynabeads ratio of 1:5, Gibco, Lithuania). The cells were cultured in RPMI 1640 supplemented with 10% fetal calf serum (FBS), 2 mM L-glutamine, 1 mM sodium pyruvate, and 1% penicillin/streptomycin.

### Transwell assay

To investigate whether cell-to-cell contact is critical for the reciprocal promotion of cytokine or immunoglobulin production during the ILC3-B-cell interaction, Transwell experiments were performed. In brief, sorted ILC3s were added into the upper chamber of a 24-well Transwell plate (6 × 10^4^ cells/well), and sorted autologous B cells were added into the bottom chamber (3 × 10^5^ cells/well) in the presence of 50 ng/mL IL-7, 50 ng/mL IL-23 and 1 μM ODN2006. The chambers were separated with a 0.4 μm pore membrane (Corning, Kennebunk). All experiments were performed in duplicate. After 4 days, the supernatants in both the upper and bottom chambers were harvested and pooled to detect cytokines and immunoglobulins using enzyme-linked immunosorbent assay (ELISA) and cytometric bead array (CBA) assays.

### Giemsa staining and multiplexed immunofluorescence staining

Tonsillar ILC3s before or after activation and CD4^+^ T cells were resuspended in RPMI-1640 containing 10% FBS. Cells were then centrifuged, placed onto slides by centrifugation and subjected to Giemsa staining. According to the manufacturer’s instructions, Ridge-Giemsa solution A was added dropwise to cells on glass slides followed by incubation for 30 s. Cells were then washed with phosphate solution for 3 min, washed with distilled water to remove excess dye, dried, and observed under a microscope. The Giemsa staining kit used in this experiment was from BASO.

Multiplexed immunofluorescence staining was performed by sequentially staining 4-µm-thick formalin-fixed, paraffin-embedded tonsil sections with standard and primary antibodies followed by application of the TSA 4-color kit (abs50012, Absinbio, Shanghai) according to the manufacturer’s instructions. The antibodies and fluorescent dyes involved were as follows: anti-CD3 (diluted at 1:200, CST, Danvers)/TSA 650, anti-CD20 (diluted at 1:200, CST, Danvers)/TSA 570, and anti-RORγt (diluted at 1:600, Abcam, Cambridge)/TSA 520. Sections were washed two times with TBST, and slides were mounted in DAPI Fluoromount-G Mounting Medium (Invitrogen). Immunofluorescence images were obtained using an LSM 710 inverted confocal microscope (Zeiss, Germany).

### Detection of cytokines using ELISA and CBA assay

The ability of ILC3s and B cells to secrete cytokines or immunoglobulins was detected using ELISA and CBA kits. The supernatants of ILC3s or B cells after stimulation, Transwell culture and coculture were collected and stored at −80 °C. IL-22 in the supernatants was detected using ELISA MAX™ Deluxe Set Human IL-22 (BioLegend, San Diego) according to the manufacturer’s instructions, and optical densities were measured using a spectrophotometer. For IL-17A, IFN-γ, total TNF, GM-CSF, IL-10, IgA, IgM, and IgG detection, corresponding CBA Flex Sets (BD Biosciences, San Diego) were used to fluorescently label the beads bound with cytokines or immunoglobulin in the supernatant, and the concentrations were quantified using a BD LSRFortessa flow cytometer and the FCVP Array v3 system.

### RNA sequencing and analysis

Tonsillar ICOS^−^ and ICOS^+^ ILC3s from one donor (*n* = 3) were sorted by flow cytometry into an Eppendorf tube containing TRIzol (Invitrogen, 1 × 10^6^ cells/mL) and quickly transferred to liquid nitrogen. Sequencing reads were obtained using the DNBSEQ-T7 platform, and RNASeq analysis was performed by the Beijing Genomics Institute. Sequencing reads were aligned to the GRCh38 human genome. To remove low-quality data, adapters were trimmed using Cutadapt 1, and low-quality bases were removed by ERNE2. To analyze differentially expressed genes (DEGs), the quality-checked reads were processed using Dr. Tom’s platform (https://biosys.bgi.com). Only protein-coding genes were considered, and gene level expression values were determined as fragments per kilobase million mapped (FPKM). All genes were analyzed with an established DEGseq analysis method (log2 fold change ≥1.5, *Q* ≤ 0.005). Kyoto Encyclopedia of Genes and Genomes (KEGG) pathway enrichment analysis was performed, and the significantly enriched terms were identified based on low *P* values.

### Statistical analysis

Student’s paired *t-*test, matched and unmatched one-way analysis of variance (ANOVA) and Tukey’s multiple comparison test were used to determine the significance of differences between ILC3s with or without activation or costimulation using Prism 6.0c (GraphPad). The *P* values are shown in the figures, and *P* ≤ 0.05 were considered significant.

## Supplementary information

Supplementary figure legends

Supplementary Figure 1

Supplementary Figure 2

Supplementary Figure 3

Supplementary Figure 4

Supplementary Figure 5

Supplementary Figure 6

Supplementary Figure 7

Supplementary Figure 8

Supplementary Table 1
