# Supplementary material for: Reciprocal costimulatory molecules control the activation of mucosal type 3 innate lymphoid cells during engagement with B cells
Source: Cell Mol Immunol. 2023 May 25;20(7):808–19. doi: 10.1038/s41423-023-01041-w (PMC10310834; doi:10.1038/s41423-023-01041-w)
Supplement: Supplementary file 1 — Supplementary figure legends [file 41423_2023_1041_MOESM1_ESM.docx]

**Supplementary Information for**

**Reciprocal Costimulatory Molecules Control the Activation of Mucosal Type 3 Innate Lymphoid Cells During Engagement with B Cells**

Xinping Lv^1,2^, Shan Zhu^1,2^, Jing Wu^1,2^, Jinfeng Shi^3^, Qiuyu Wei^2^, Tete Li^2,4^, Ning Yang^2^, Chunyan Liu^2^, Lingli Qi^2,5^, Guoxia Zang^2^, Hang Cheng^2,6^, Zhiguang Yang^7^, Chengyan Jin^8^, Yusheng Wang^3^, Jiuwei Cui^1^, Hideki Ueno^9,10^, Yong-Jun Liu^2^*, Jingtao Chen^1,2^*

**Affiliations:**

^1^Cancer Center, First Hospital of Jilin University, Changchun, Jilin, 130021, China.

^2^Laboratory for Tumor Immunology, First Hospital of Jilin University, Changchun, Jilin, 130061, China.

^3^Department of Otolaryngology Head and Neck surgery, First Hospital of Jilin University, Changchun, China.

^4^Department of Translational Medicine, Changchun GeneScience Pharmaceuticals Co., Ltd., Changchun, China.

^5^Department of Pediatric Gastroenterology, First Hospital of Jilin University, Changchun, Jilin, 130021, China.

^6^Department of Pediatrics, First Hospital of Jilin University, Changchun, Jilin, 130021, China.

^7^Department of Thoracic surgery, First Hospital of Jilin University, Changchun, China.

^8^Department of Thoracic surgery, Second Hospital of Jilin University, Changchun, Jilin, 130041, China.

^9^Department of Immunology, Graduate School of Medicine, Kyoto University, Kyoto, Japan.

^10^ASHBi Institute for the Advanced Study of Human Biology, Kyoto University, Kyoto, Japan.

*Corresponding author. Email: jtchen@jlu.edu.cn. Telephone: +86-431-88783130 (J. Chen); yjliuanderson@jlu.edu.cn. Telephone: +86-431-88783144 (Y.-J. Liu).

**FIGURE LEGENDS**

**Supplementary Fig. 1. Gating strategy and phenotypes of ILC subsets. (a)** In tonsillar lymphocytes (CD45^+^), through progressive gating, total innate lymphoid cells (ILCs) were defined as CD3^−^ Lineage^−^ (CD3 CD19 CD20 CD14 CD94 CD34 CD1a CD11c CD123 TCRα/β TCRγ/δ FcεRIα) CD127^+^ cells, ILC1s (CRTH2^−^ CD117^−^, green frame), ILC2s (CRTH2^+^ CD117^−/+^, blue frame) and ILC3s (CRTH2^−^ CD117^+^, red frame). **(b, c)** Histogram comparison of ROR-γt (b) and inducible T cell costimulator ligand (ICOS) expression (c) in tonsillar ILC1s (green), ILC2s (blue), ILC3s (red) and Fluorescence Minus One (FMO) control (filled gray). Data represent three donors. **(d)** Flow cytometric analysis of ICOS and NKp44 coexpression on ILC3s from the tonsil, distal and normal lung of donor with lung cancer and peripheral blood of healthy donor.

**Supplementary Fig. 2. Purification, characterization, and activation of ILC3s. (a, b)** Verification of ILC3s isolated from tonsil by MACS plus FACS. **(a)** Sorted ILC3s were reanalyzed through progressive gating according to the gating strategy in Figure S1a. Purity of ILC3s were calculated by multiplying of percentages of each gating. **(b)** Histogram of PE-conjugated T-bet (green), GATA3 (blue), ROR-γt (blue) and Fluorescence Minus One (FMO) control (filled gray) of sorted ILC3s after transcription factor staining. **(c)** ILC3s were sorted from peripheral blood of healthy donor-derived lymphocytes according to the gating strategy in Figure S1a. Median fluorescence intensity (MFI) of ICOS on PB-derived ILC3s stimulated by IL-1, IL-2, IL-1β and IL-23 for 7 days in vitro. **(d, e)** Purified ILC3s from the same donor were stimulated by IL-2+IL-7 or IL-2+IL-7+IL-1β+IL-23 (n = 3-8). **(d)** Representative flow plots and quantification of viable (upper), ki-67^+^ (middle), CD25^+^ ILC3s (lower) (n = 3-6). **(e)** Concentration of IL-22, IL-17A, total TNF, GM-CSF and IFN-γ in the supernatants of ILC3s after stimulation as determined by enzyme linked immunosorbent assay (ELISA) (n = 5-8). Error bars indicate the mean ± standard error of mean (SEM), and two-tailed P-values were determined using a Student’s paired t-test in d and e.

**Supplementary Fig. 3. ICOS^+^ ILC3s differ with ICOS^−^ ILC3 at the transcriptome level. (a-b)** Enrichment analysis of Kyoto Encyclopedia of Genes and Genomes (KEGG) pathways (a) and Heatmaps (b) of tonsillar ICOS^−^ and ICOS^+^ ILC3 differentially expressed genes (DEGs). DEGseq analysis (log 2 of fold change ≥ 1.5, Q-value ≤ 0.005) was used to test for DEGs (n = 3). Results are shown as a scatter plot of Z-score (row direction) for fragments per kilobase million mapped (FPKM).

**Supplementary Fig. 4. ICOS^+^ ILC3s differ with ICOS^−^ ILC3 and ICOS^−/+^ CD4^+^ T cells functionally. (a)** Functional comparison of tonsillar ICOS^−^ and ICOS^+^ ILC3s as well as tonsillar ICOS^−^ and ICOS^+^ CD4^+^ T cells. ICOS^−/+^ ILC3s and ICOS^−/+^ CD4^+^ T cells sorted from the same donor were incubated at the same cell concentration and stimulated by IL-7+IL-2+IL-1β+IL-23 (for ILC3s) or anti-CD3/CD28 (for CD4^+^ T cells) in vitro. At day 7, IL-22, IL-17A, total TNF, GM-CSF and IFN-γ in the supernatants were determined by ELISA and CBA assays (n = 4–9). **(b)** CBA assay of IL-10 in the supernatants of ICOS^−/+^ ILC3s and ICOS^−/+^ CD4^+^ T cells after activation on days 4 and 7 (n = 6–9). Error bars indicate the mean ± SEM. Statistical significance was determined using an unmatched one-way ANOVA and Tukey’s multiple comparison test.

**Supplementary Fig. 5. ILC3s do not acquire free ICOSL for ICOS costimulation. (a)** ILC3s isolated from tonsil by FACS were incubated in rsICOSL precoated wells or not (control) in the presence of IL-7, IL-1β and IL-23 for 4 days. Representative flow plots and quantification of NKp44^+^ (left) and CD25^+^ ILC3s (right). Data represent nine (a left) and six (a right) experiments. **(b)** ELISA and CBA assays of cytokines in the supernatants of ILC3s incubated in the presence or absence (control) of free rsICOSL with suboptimal stimuli (IL-7+IL-1β+IL-23) of ILC3s for 4 days (n = 3–6). **(c)** ILC3s were cocultured with the CD32/ICOSL-expressing cell line or parental CD32-expressing cell line (control) at a ratio of 4:1 for 4 days. Representative flow plots of IL-22^+^ ILC3s (n = 3). Error bars indicate the mean ± SEM, and two-tailed P-values were determined using a Student’s paired t-test.

**Supplementary Fig. 6. Predominant B cells promote survival with ILC3s reciprocally in the tonsil. (a)** FACS of tonsillar lymphocytes (CD45^+^) using multiple surface biomarkers identified different immune cells and calculated percentages of CD45^+^ cells. CD4^+^ T cells (CD3^+^ CD4^+^), CD8^+^ T cells (CD3^+^ CD4^−^), B cells (CD19^+^), mDCs (CD3^−^ CD4^+^ CD123^−^ CD11c^+^), pDCs (CD3^−^ CD4^+^ CD123^+^ CD11c^−^), monocytes (CD19^−^ CD14^+^) and NK cells (CD14^−^ CD56^+^). Data represent five donors. **(b)** ILC3s and B cells isolated from the same donor were cocultured at ratios of 1:1, 1:5, 1:10 and 1:15 (ILC3: B cell, quantitative cell number of ILC3s, and variable cell numbers of B cells, respectively) in the presence of IL-7, IL-23 and ODN2006 for 4 days. Flow cytometric analysis of the percentages of live B cells of B cells only (first row) and B cells cocultured with ILC3s (second row), as well as the percentages of live ILC3s, of ILC3s only (third row), and ILC3s cocultured with B cells (last row).

**Supplementary Fig. 7. Effects of ILC3 activators on B cells and impacts of ODN2006 on ILC3s. (a)** CBA assays of IgA, IgM and IgG in the supernatant of purified tonsillar B cells stimulated by ILC3 activators (IL-7, IL-2, IL-1β and IL-23) and ODN2006 for 4 days. **(b-d)** Tonsillar ILC3s and B cells (CD3^−^ CD19^+^) sorted from the same donor were cocultured at ratio 1:15 in the presence of IL-7+IL-2 or IL-7+IL-2+IL-1β+IL-23 for 4 days. **(b)** Absolute cell number of ILC3s (n = 7). **(c)** Representative flow plots and quantification of CD69^+^ ILC3s (n = 5). **(d)** Representative flow plots of IL-22^+^ ILC3s (n = 3). **(e)** Representative flow plots and percentages of viable (upper), ki-67^+^ ILC3s (middle) and ICOS^+^ NKp44^+^ ILC3s (lower) stimulated with ODN2006 for 7 days. **(f)** ELISA and CBA assays of cytokines (IL-22, IL-17A, total TNF, GM-CSF and IFN-γ) in the supernatant of ILC3s incubated in the presence of absence of ODN2006 for 7 days. **(g)** ICOS^−/+^ ILC3s and ICOS^−/+^ CD4^+^ T cells sorted from the same donor were incubated at the same cell concentration and stimulated by IL-7+IL-2+IL-1β+IL-23 (for ILC3s) or anti-CD3/CD28 (for CD4^+^ T cells) in vitro. CBA assay of IL-21 in the supernatant of ICOS^−/+^ ILC3s and ICOS^−/+^ CD4^+^ T cells after activation on day 4 (left) and 7 (right) (n = 3). Error bars mean ± SEM, and two-tailed P-values in (c) were determined using a Student’s paired t-test.

**Supplementary Fig. 8. Reciprocal costimulatory molecules control the activation of mucosal ILC3s during engagement with B cells.** In human lymphoid tissues, ICOS is found expressed on a portion of ILC3s and functions as one of the secondary activation signals that contributes to ILC3 survival, proliferation, and cytokine production, including IL-22 and IL-17A in human lymphoid tissues. Some ILC3s colocalize with nearby B cells in lymphoid tissue. The interaction of ILC3s and colocalized autogenous B cells in lymphoid tissue can functionally promote the production of cytokines (IL-22, IL-17A, TNF, GM-CSF, and IFN-γ) by ILC3s and production of T cell-independent IgA, IgM, and IL-10 by B cells. In addition, this interaction of ILCs and B cells promotes the survival and proliferation of ILC3s and B cells. This reciprocal activation between ILC3s and B cells is partially involved with ICOS and CD40-mediated interactions on the cell surface, and some other factors such as B cell activation factor (BAFF).

**Supplementary Table 1. Differentially expressed genes between tonsillar ICOS^−^ ILC3 and ICOS^+^ ILC3s**

[**Differentially expressed genes (ICOS−ILC3 vs. ICOS+ILC3s).xlsx**](../../../../../Differentially%20expressed%20genes%20(DEGseq%20FC1.5%20Q0.05).xlsx)

**Supplementary Table 2. Antibodies used in flow cytometry**

| **No.** | **Reactivity** | **Antibody** | **Conjugation** | **Clone No.** | **Source** |
| --- | --- | --- | --- | --- | --- |
| 1 | Human | CD45 | APC/Cyanine7 | 2D1 | BioLegend |
| 2 | Human | CD45 | APC | HI30 | BD Biosciences |
| 3 | Human | CD3 | FITC | HIT3a | BioLegend |
| 4 | Human | CD3 | Alexa Fluor 700 | SK7 | BioLegend |
| 5 | Human | CD3 | PerCP-Cy5.5 | UCHT1 | BD Biosciences |
| 6 | Human | CD3 | BV421 | SK7 | BD Biosciences |
| 7 | Human | CD19 | APC-H7 | HIB19 | BD Biosciences |
| 8 | Human | CD19 | FITC | 2H7 | BioLegend |
| 9 | Human | CD20 | PE/Cyanine7 | 2H7 | BioLegend |
| 10 | Human | CD4 | PE-CF594 | RPA-T4 | BD Biosciences |
| 11 | Human | CD4 | PE-Cy7 | G44-26 | BD Biosciences |
| 12 | Human | Lineage Cocktail 3 | FITC |  | BD Biosciences |
| 13 | Human | CD1a | FITC | HI149 | BioLegend |
| 14 | Human | CD94 | FITC | DX22 | BioLegend |
| 15 | Human | CD11c | FITC | 3.9 | BioLegend |
| 16 | Human | CD11c | V450 | B-ly6 | BD Biosciences |
| 17 | Human | CD34 | FITC | 581 | BioLegend |
| 18 | Human | CD123 | FITC | 6H6 | BioLegend |
| 19 | Human | TCRγ/δ | FITC | B1 | BioLegend |
| 20 | Human | TCRα/β | FITC | IP26 | BioLegend |
| 21 | Human | FcεRIα | FITC | AER-37 (CRA-1) | BioLegend |
| 22 | Human | CD127 | Percp-Cy5.5 | A019D5 | BioLegend |
| 23 | Human | CRTH2(CD294) | PE-Cy7 | BM16 | BioLegend |
| 24 | Human | CD117 (c-kit) | Dazzle 594 | 104D2 | BioLegend |
| 25 | Human | CD117 (c-kit) | Bv605 | 104D2 | BioLegend |
| 26 | Human | RORγt | PE | Q21-559 | BD Biosciences |
| 27 | Human | RORγt | BV421 | Q21-559 | BD Biosciences |
| 28 | Human | GATA3 | PE | 16E10A23 | BioLegend |
| 29 | Human | T-bet | PE | 4B10 | BioLegend |
| 30 | Human | ICOS (CD278) | Bv421 | C398.4A | BioLegend |
| 31 | Human | ICOS (CD278) | AF647 | C398.4A | BioLegend |
| 32 | Human | ICOS (CD278) | APC | ISA-3 | eBioscience |
| 33 | Human | ICOS (CD278) | PE | C398.4A | BioLegend |
| 34 | Human | ICOSL (B7-H2, CD275) | PE | 2D3 | BioLegend |
| 35 | Human | CD40L (CD154) | PE | 24-31 | BioLegend |
| 36 | Human | NKp30 (CD337） | PE | P30-15 | BioLegend |
| 37 | Human | NKp44 (CD336) | AF647 | p44-8 | BD Biosciences |
| 38 | Human | NKp46 (CD335) | AF700 | 9E2 | BioLegend |
| 39 | Human | CCR6 (CD196) | PE | G034E3 | BioLegend |
| 40 | Human | CCR7 (CD197) | PE | G043H7 | BioLegend |
| 41 | Human | CCR7 (CD197) | PE | 3D12 | BD Biosciences |
| 42 | Human | CCR9 (CD199) | PE | L053E8 | BioLegend |
| 43 | Human | CXCR3 (CD183) | PE | G025H7 | BioLegend |
| 44 | Human | CXCR5 (CD185) | PE | J252D4 | BioLegend |
| 45 | Human | CD28 | PE | CD28.2 | BD Biosciences |
| 46 | Human | PD-1 (CD279) | PE | MIH4 | BD Biosciences |
| 47 | Human | PD-L1 (CD274) | PE | 29E.2A3 | BioLegend |
| 48 | Human | CD69 | BV605 | FN50 | BD Biosciences |
| 49 | Human | ki-67 | PE/Dazzle 594 | Ki-67 | BioLegend |
| 50 | Human | CD25 | Alexa Fluor 700 | BC96 | BioLegend |
| 51 | Human | CD14 | APC/Cyanine7 | M5E2 | BioLegend |
| 52 | Human | CD56 | Alexa Fluor 700 | HCD56 | BioLegend |
| 53 | Human | IL-17A | Alexa Fluor 700 | BL168 | BioLegend |
| 54 | Human | IL-22 | PE | 22URTI | eBioscience |
| 55 | Human | CD32 | PE-Cy7 | FUN-2 | BioLegend |
| 56 | Human | CD40 | FITC | HB14 | BioLegend |
| 57 | Human | Mouse IgG2b, κ | PE | MPC-11 | BioLegend |
