# Supplementary figures and images for: Reciprocal costimulatory molecules control the activation of mucosal type 3 innate lymphoid cells during engagement with B cells
Source: Cell Mol Immunol. 2023 May 25;20(7):808–19. doi: 10.1038/s41423-023-01041-w (PMC10310834; doi:10.1038/s41423-023-01041-w)

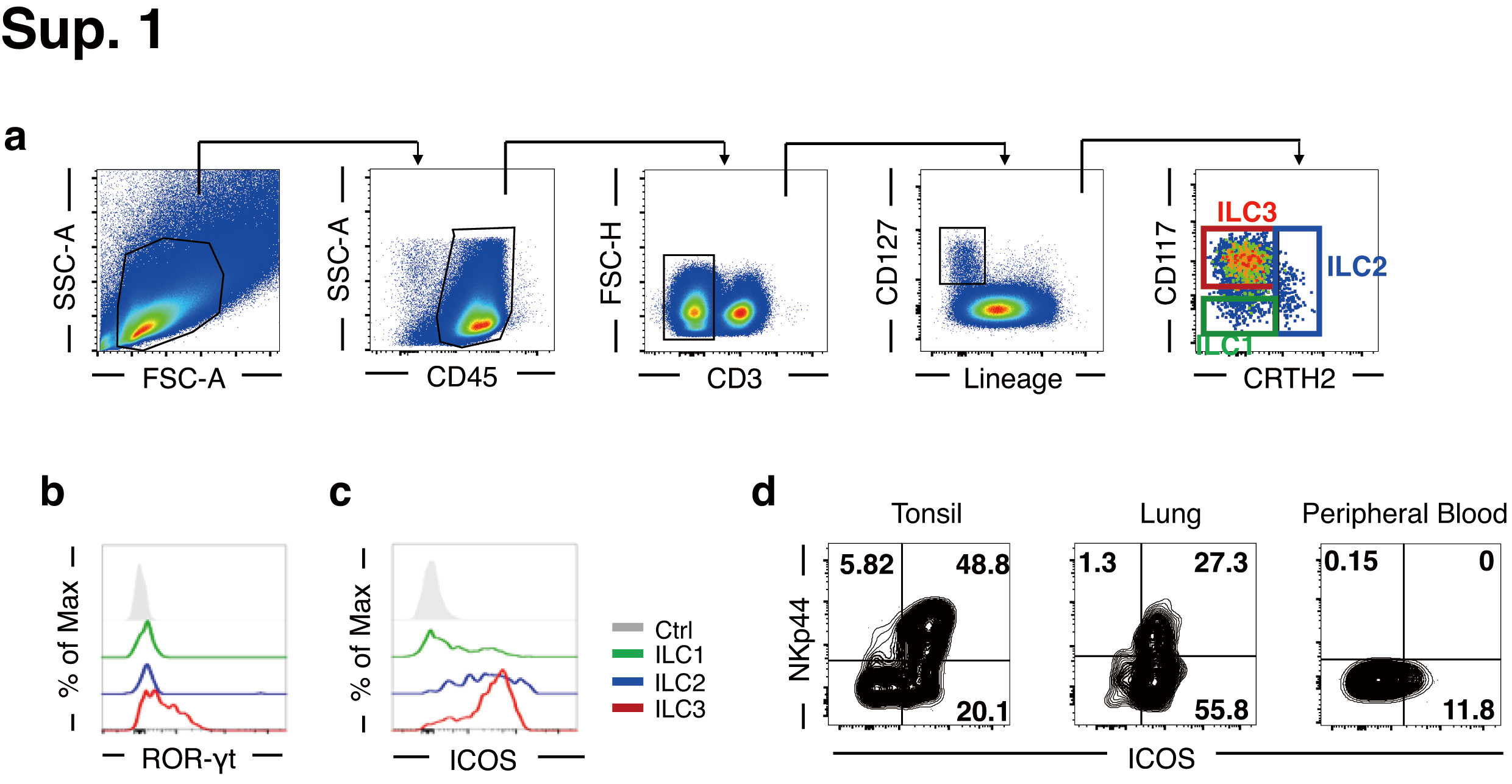

Supplement: Supplementary file 2 — Supplementary Figure 1 [file 41423_2023_1041_MOESM2_ESM.jpg]

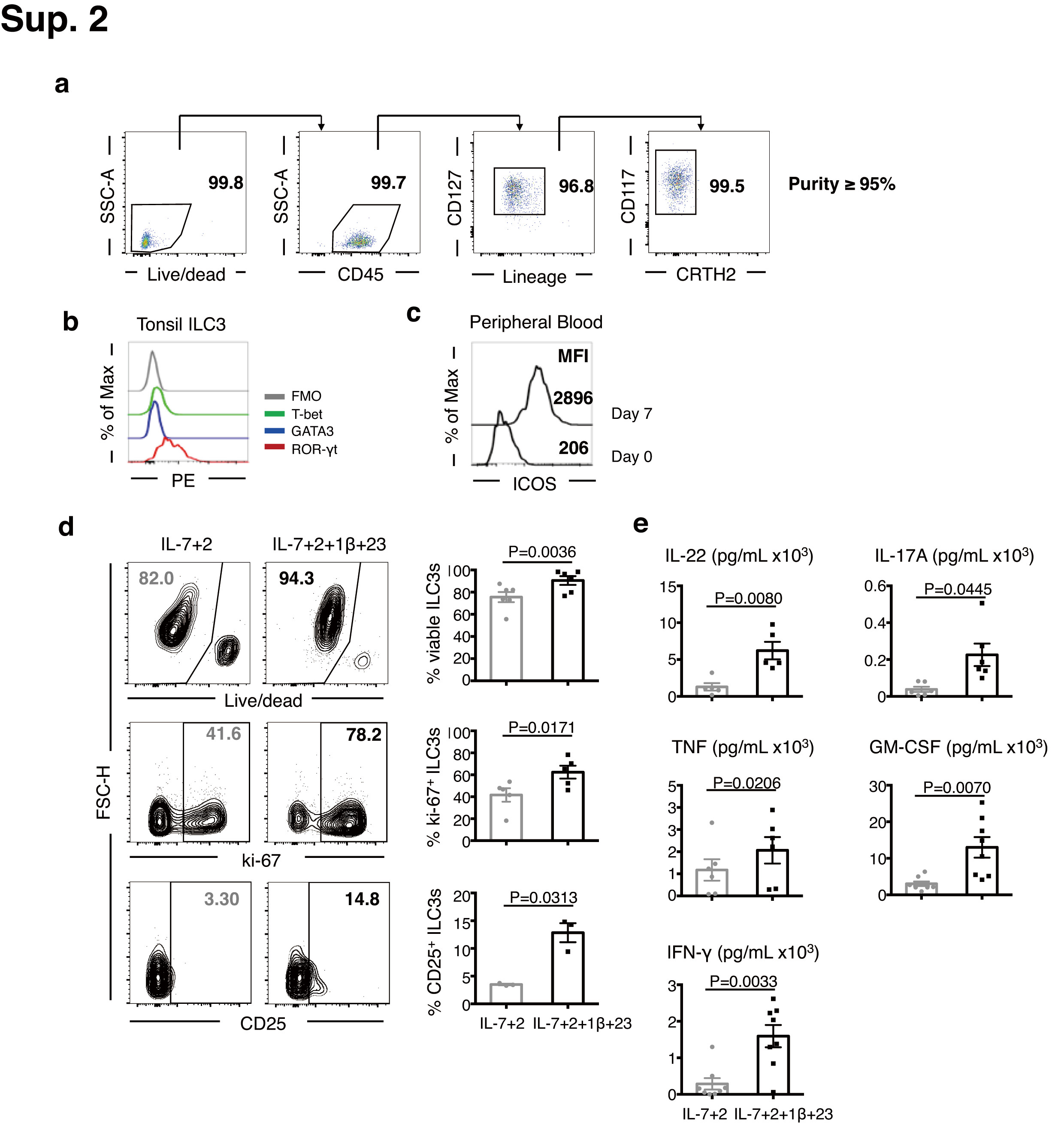

Supplement: Supplementary file 3 — Supplementary Figure 2 [file 41423_2023_1041_MOESM3_ESM.jpg]

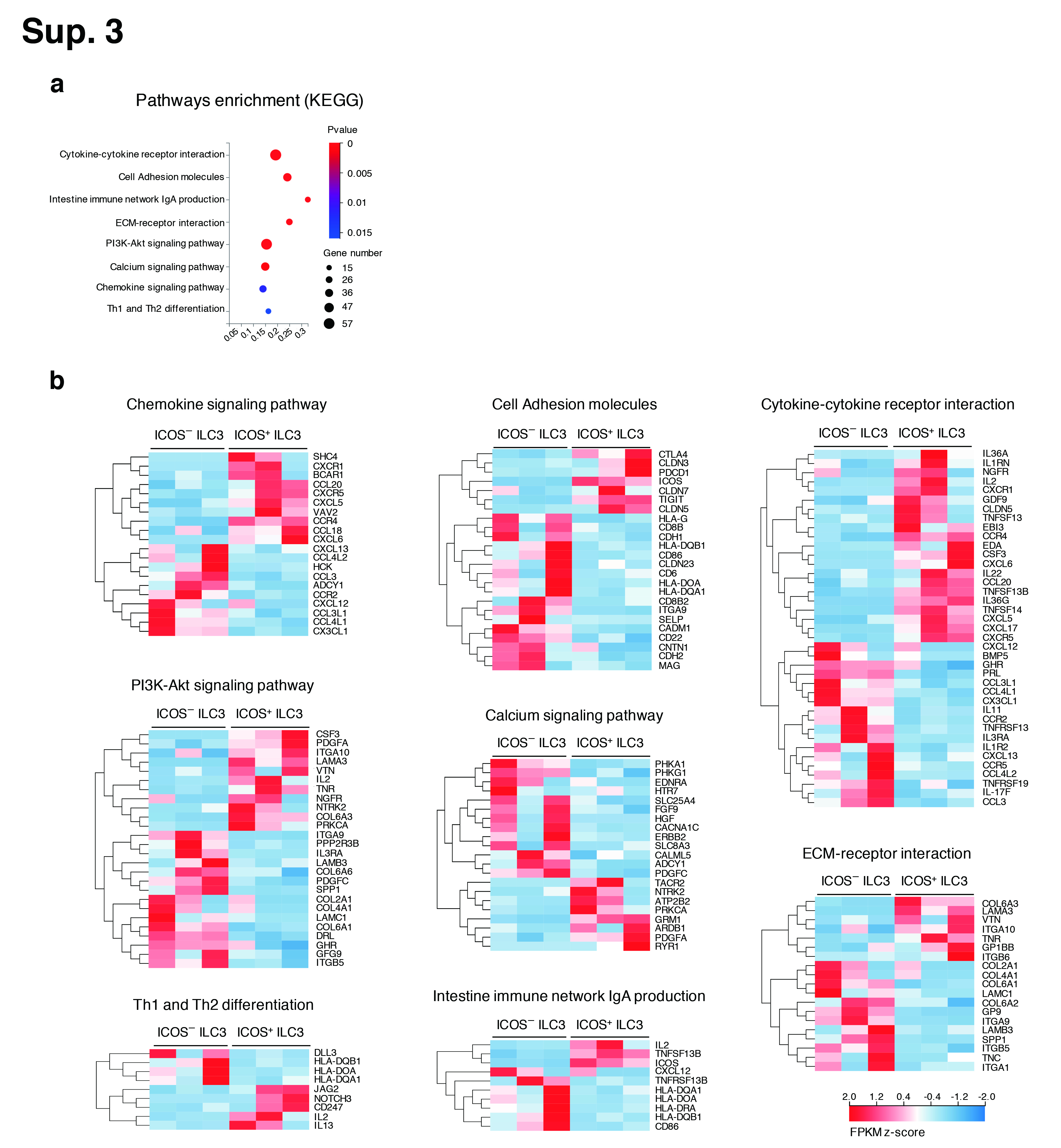

Supplement: Supplementary file 4 — Supplementary Figure 3 [file 41423_2023_1041_MOESM4_ESM.jpg]

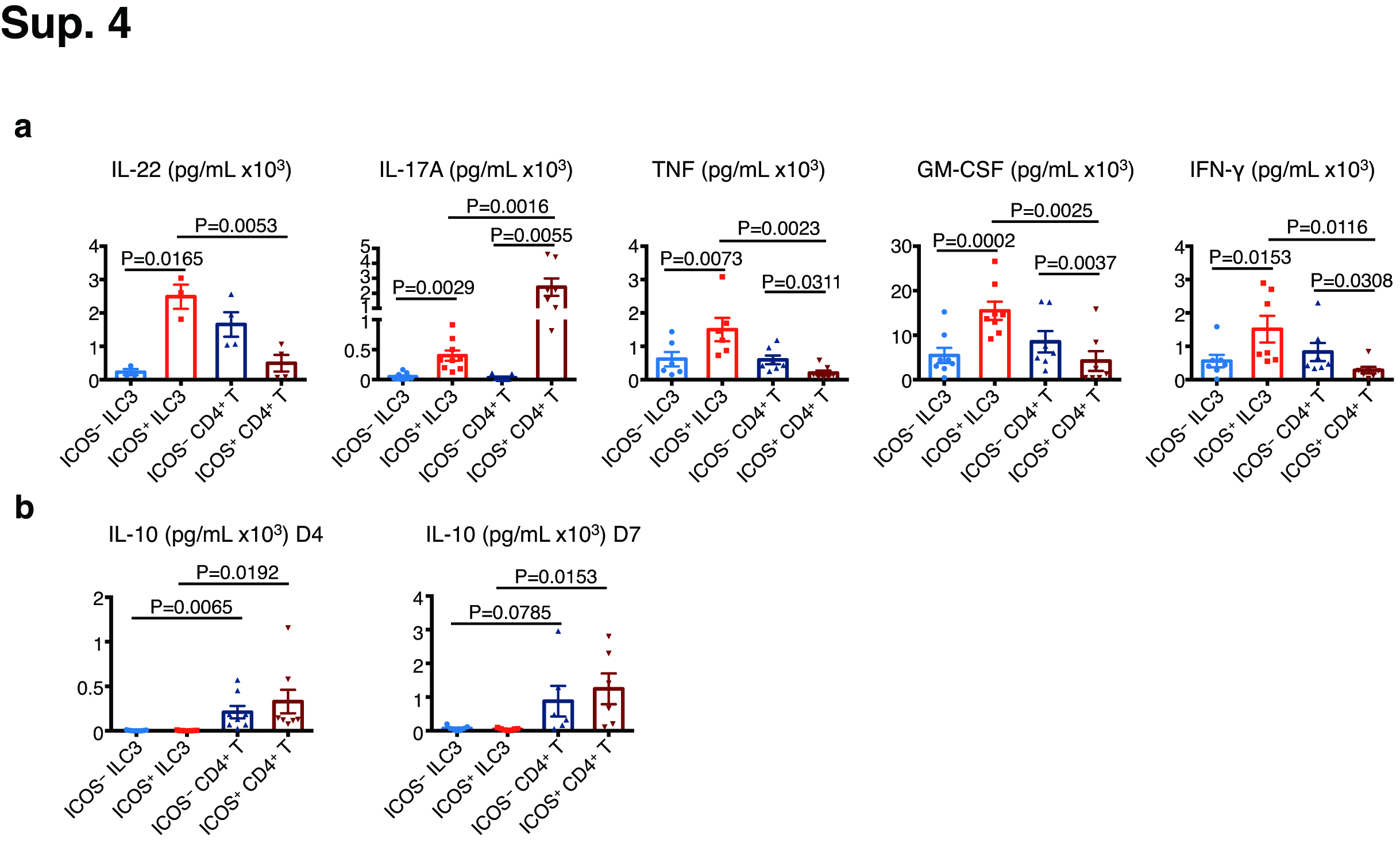

Supplement: Supplementary file 5 — Supplementary Figure 4 [file 41423_2023_1041_MOESM5_ESM.jpg]

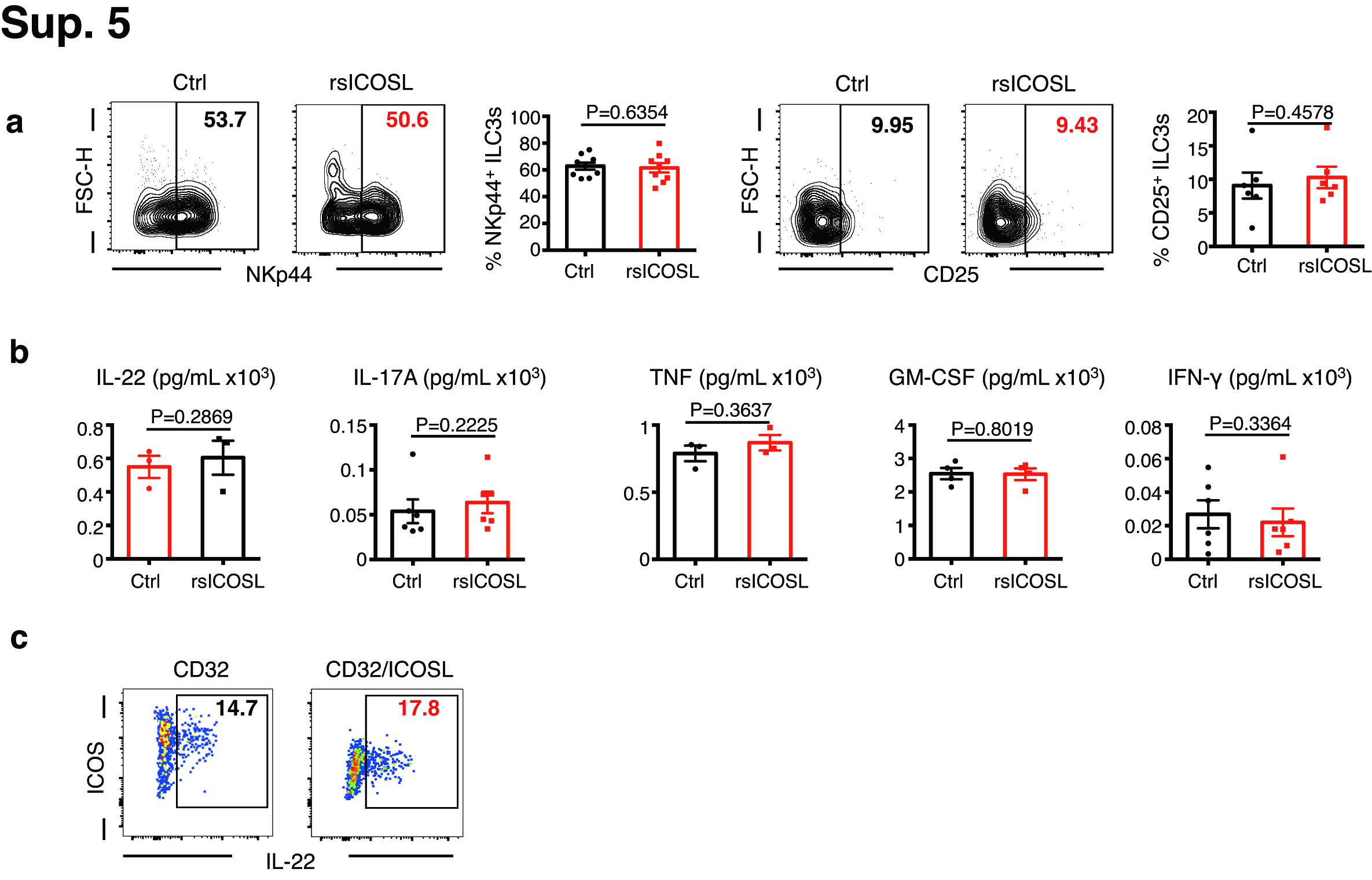

Supplement: Supplementary file 6 — Supplementary Figure 5 [file 41423_2023_1041_MOESM6_ESM.jpg]

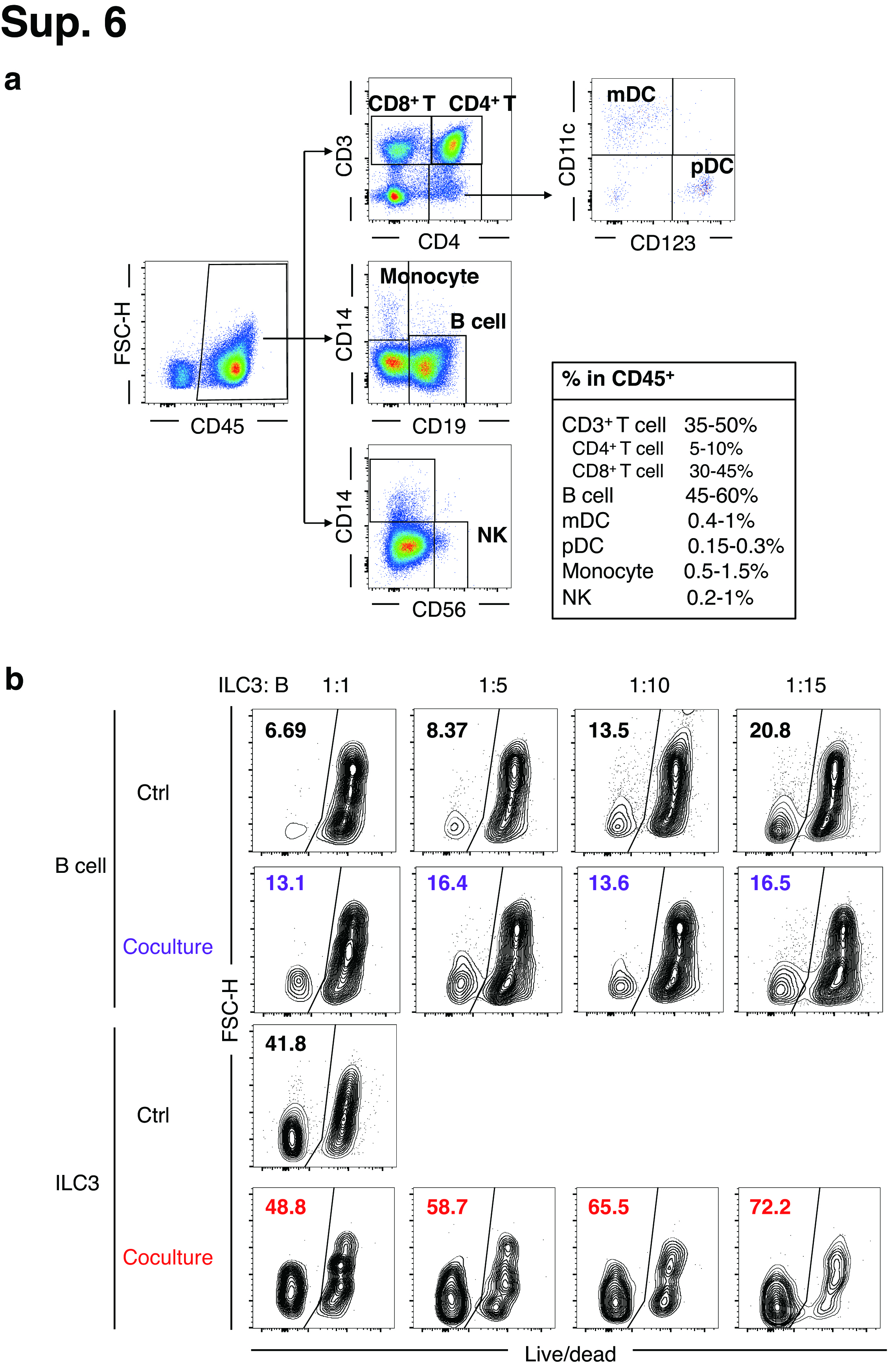

Supplement: Supplementary file 7 — Supplementary Figure 6 [file 41423_2023_1041_MOESM7_ESM.jpg]

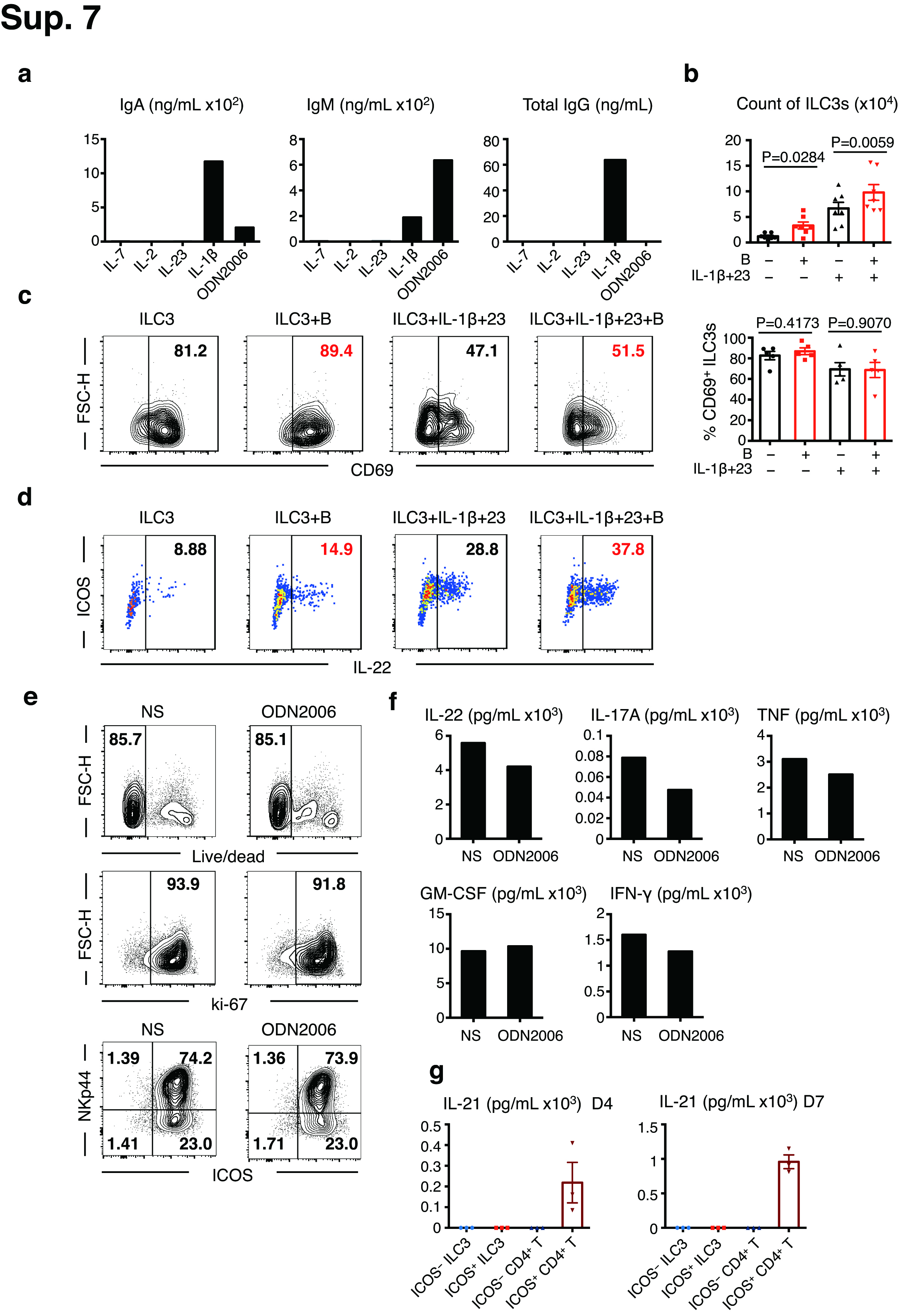

Supplement: Supplementary file 8 — Supplementary Figure 7 [file 41423_2023_1041_MOESM8_ESM.jpg]

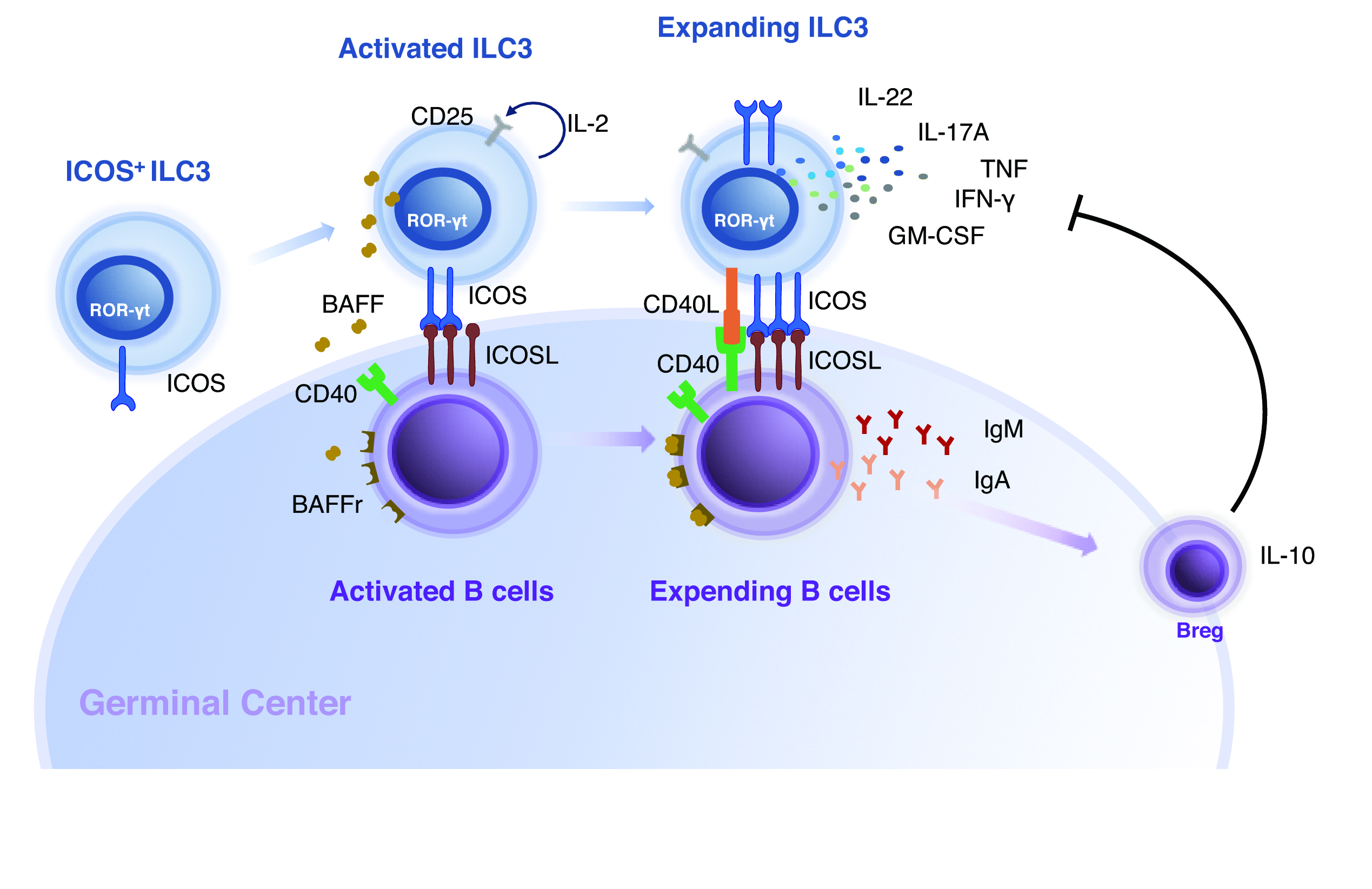

Supplement: Supplementary file 9 — Supplementary Figure 8 [file 41423_2023_1041_MOESM9_ESM.jpg]
